# Cleavage and activation of LIM kinase 1 as a novel mechanism for calpain 2-mediated regulation of nuclear dynamics

**DOI:** 10.1038/s41598-021-95797-8

**Published:** 2021-08-11

**Authors:** L. Rodríguez-Fernández, S. Company, R. Zaragozá, J. R. Viña, E. R. García-Trevijano

**Affiliations:** 1 Departamento de Bioquímica y Biología Molecular, Facultad de Medicina, Universidad de Valencia, Avda. Blasco Ibañez, 15, 46010 Valencia, Spain; 2 Fundación Investigación Hospital Clínico-INCLIVA, Valencia, Spain; 3 Departamento de Anatomía y Embriología Humana, Facultad de Medicina, Universidad de Valencia, Valencia, Spain

**Keywords:** Cancer, Cell biology

## Abstract

Calpain-2 (CAPN2) is a processing enzyme ubiquitously expressed in mammalian tissues whose pleiotropic functions depend on the role played by its cleaved-products. Nuclear interaction networks, crucial for a number of molecular processes, could be modified by CAPN2 activity. However, CAPN2 functions in cell nucleus are poorly understood. To unveil CAPN2 functions in this compartment, the result of CAPN2-mediated interactions in cell nuclei was studied in breast cancer cell (BCC) lines. CAPN2 abundance was found to be determinant for its nucleolar localization during interphase. Those CAPN2-dependent components of nucleolar proteome, including the actin-severing protein cofilin-1 (CFL1), were identified by proteomic approaches. CAPN2 binding, cleavage and activation of LIM Kinase-1 (LIMK1), followed by CFL1 phosphorylation was studied. Upon CAPN2-depletion, full-length LIMK1 levels increased and CFL1/LIMK1 binding was inhibited. In addition, LIMK1 accumulated at the cell periphery and perinucleolar region and, the mitosis-specific increase of CFL1 phosphorylation and localization was altered, leading to aberrant mitosis and cell multinucleation. These findings uncover a mechanism for the role of CAPN2 during mitosis, unveil the critical role of CAPN2 in the interactions among nuclear components and, identifying LIMK1 as a new CAPN2-target, provide a novel mechanism for LIMK1 activation. CFL1 is crucial for cytoskeleton remodeling and mitosis, but also for the maintenance of nuclear structure, the movement of chromosomes and the modulation of transcription frequently altered in cancer cells. Consequently, the role of CAPN2 in the nuclear compartment might be extended to other actin-associated biological and pathological processes.

## Introduction

Calpain-2 (CAPN2) is a Ca^2+^-dependent cysteine protease ubiquitously expressed in mammalian tissues^1^. In contrast to other proteases, the result of CAPN2 enzymatic activity is not substrate degradation, but a cleaved-substrate with different functions, distribution, interacting proteins or ways of regulation^2,3^. Accordingly, the plethora of physiological or pathological functions described for CAPN2 are related to the role played by its end-products^2,4–10^. From that point of view, CAPN2 could be considered as a regulatory node for different signaling pathways. Therefore, CAPN2-targets should be identified in order to determine CAPN2 functions in a particular cellular context. This is not an easy task taking into account the following considerations: (*i*) CAPN2 proteolyzes in vitro a large number of substrates that are not necessarily cleaved in vivo^3,11^; (*ii*) CAPN2-mediated cleavage of substrates is not dependent on their primary structure or post-translational modification^11^; and, (*iii*) CAPN2 expression and factors related to the modulation of its enzymatic activity^2,3,12^ will play an undoubting role, but do not provide information on the context-dependent functions of CAPN2.

Current models to explain the multiple functions of CAPN2 point to its subcellular distribution. Indeed, the localization of CAPN2 in a cellular compartment according to the type of biological process, would limit its access to specific substrates^5,7–10,13,14^. Following this rational, the challenge to establish CAPN2 functions would be the identification of isoform-specific substrates of CAPN2 in a given subcellular compartment.

The role of CAPNs at cell membranes or in the cytosolic compartment has been largely studied in different cell types to explain cell migration, cell adhesion or cell death^4–8,10^. However, CAPN2 functions in the nuclear compartment have not been completely elucidated. Several studies have shown that in proliferating cells, CAPN2 is mainly localized in the nucleus^14–16^, while in differentiated quiescent cells CAPN2 is restricted to the cytosol^15^. Moreover, nuclear localization of CAPN2 has been associated to high expression levels and active mitosis in embryonic stem cells as well as in 8-cell embryos^14^. In this sense, chromosome misalignment and aberrant mitosis has been reported in CAPN2-depleted HeLa cells^17,18^. Although these data indicate that CAPN2 is most probably involved in the modulation of cell cycle or mitosis, CAPN2-targets and mechanisms underlying these effects remain elusive. Our group previously showed that CAPN2 is localized in the nucleolus of colorectal cancer cells participating in rRNA biogenesis under growth-adverse conditions^13^; however, those CAPN2-targets responsible for this or other effects were not identified. This is an important issue, since components of a particular subnuclear structure can be eventually sorted to other substructures where they are modified, retained and/or released depending on the stage of cell cycle or type of stimulus^19,20^.

The dynamic changes in the interaction among nuclear components are crucial for gene expression, cell proliferation, DNA repair or cell death^19^. Post-translational modifications change the localization and interactions networks. Presumably, proteins processed by CAPN2 would change the nuclear interaction networks. In this study we sought to identify the CAPN2-mediated components of these interactions in the cell nuclei of proliferating cells.

## Results and discussion

### Subnuclear distribution of CAPN2 in proliferating breast cancer cell lines

High levels of CAPN2 have been associated to CAPN2 nuclear localization in proliferating cells^14^. We previously described that luminal cells (MCF-7 and BT-474) express lower levels of CAPN2 than triple negative breast cancer cells (MDA-MB-231 and MDA-MB-468)^8^. Nuclear distribution of CAPN2 was studied in asynchronous cultured cells by immunofluorescence staining and immunoblotting. Different cell lines were selected according to their CAPN2 levels^8^.

**Figure 1:**
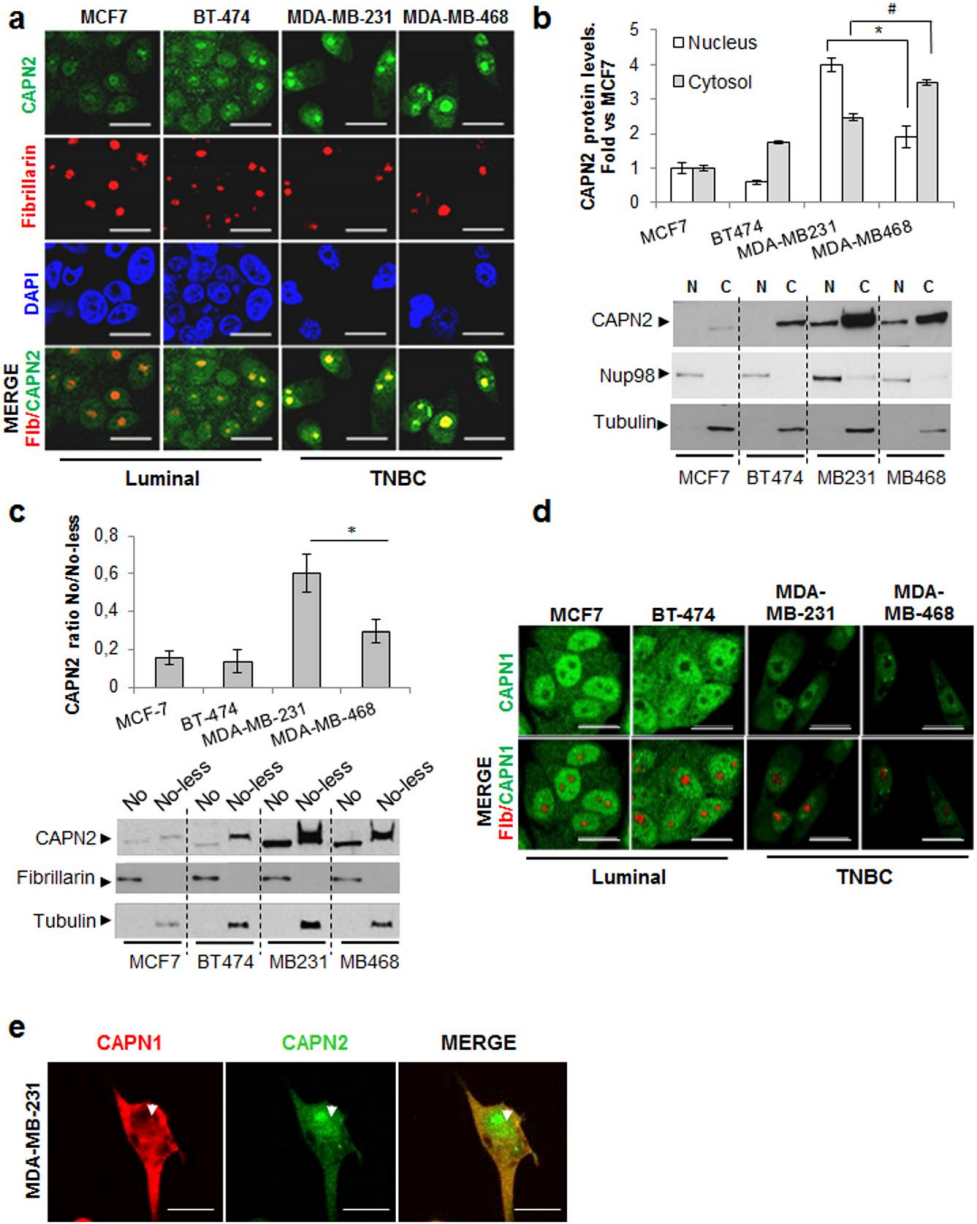
Subnuclear distribution of CAPN2 in breast cancer cell lines. (**a**) Confocal images of CAPN2 (green), fibrillarin (red), DAPI (blue) and merge in BCC lines. Scale bars 25 μm. (**b**) Subcellular distribution of CAPN2 analyzed by western blot in cytosolic (C) and nuclear (N) fractions. Data (n ≥ 3) were quantified, normalized with Nup98 (N) or tubulin (C) and plotted as mean fold ± SEM *vs.* MCF-7 cells. *p ≤ 0.01 nuclear and #p ≤ 0.01 cytosolic fractions. (**c**) Subcellular distribution of CAPN2 in nucleolar (No) and Nucleolar-less (No-less) fractions analyzed by western blot. Data (n ≥ 3) were quantified, normalized with fibrillarin (No) or tubulin (No-less) and plotted as mean No/No-less ratio vs. MCF7 cells, *p ≤ 0.01. (**d**) Immunofluorescence staining of CAPN1 (green) and fibrillarin (red) in BCC lines. Scale bars 25 μm. (**e**) Colocalization of CAPN1 (red) and CAPN2 (green) by staining with anti-mouse CAPN1 and anti-rabbit CAPN2 antibodies in MDA-MB-231 cells. Scale bars 15 μm. Juxtaposed images from different lanes in the same blot are separated by dotted lines in (**b**, **c**). Full-length blots are shown in Supplementary Fig. S14. Representative images of all cells are shown in (**a**, **d**, **e**).

In agreement with our previous reports in colorectal cancer cells^13^, CAPN2 staining was detected in the nucleoplasm of all cell lines studied (Fig. 1A). However, nuclear CAPN2 levels were higher in triple negative (TNBC) cells, those with the highest metastatic potential. Immunoblots of cytosolic and nuclear fractions also showed that, in addition to the cytosol, CAPN2 was strongly accumulated in the nuclear compartment of TNBC cells (Fig. 1B). These data are consistent with a preferential localization of CAPN2 in nuclei of undifferentiated cells^14–16^.

Colocalization of CAPN2 and the nucleolar marker fibrillarin demonstrated its strong accumulation in nucleoli of all cell lines (Fig. 1A). Subnuclear distribution of CAPN2 was also analyzed in nucleolar (No) and nucleolar-less fractions (No-Less, containing whole cell extracts except nucleoli) by western blot (Fig. 1C). CAPN2 was detected in nucleolar fractions from all cell lines. However, the nucleolar/nucleolar-less (No/No-less) ratio of CAPN2 was dramatically higher in TNBC than in luminal cell lines.

CAPN1 is the other isoform ubiquitously expressed in mammalian tissues^1^. To confirm the isoform-specific distribution of CAPN2 in breast cancer cells (BCCs), the subnuclear distribution of CAPN1 was also analyzed by confocal microscopy (Fig. 1D). CAPN1 was spread throughout the nucleoplasm of BCCs without any obvious enhancement of a particular region. Luminal cells showed higher levels of nuclear CAPN1 than TNBC cells. The analysis of CAPN1 in subcellular fractions by western blot showed the same results (Supplementary Fig. S1). Finally, double staining with CAPN1 and CAPN2 antibodies showed a limited colocalization of both isoforms in nuclei of MDA-MB-231 cells. CAPN2, but not CAPN1, was strongly accumulated in nucleoli and perinucleolar region of MDA-MB-231 cells (Fig. 1E).

Thus, it can be concluded that nuclear abundance of CAPN2 seems to be dependent on its overall expression levels according to the breast cancer subtype. In addition, both CAPNs show a differential subnuclear distribution in BCCs suggesting an isoform-specific role for nuclear CAPN2.

The composition of the nucleolus and perinucleolar region is subjected to dynamic changes throughout the cell cycle^19,20^. Proteins are sequestered in the nucleolus to control its activity in the nucleoplasm or the activity and stability of downstream targets under different types of stimuli^21,22^. We could speculate that CAPN2 would be retained in a particular subnuclear localization depending on its abundance.

### Relationship between abundance and nucleolar localization of CAPN2

To explore whether abundance of nuclear CAPN2 is the key determinant for its nucleolar accumulation, CAPN2 was either, ectopically overexpressed or downregulated in MCF-7 and MDA-MB-231, respectively (Supplementary Fig. S2).

**Figure 2:**
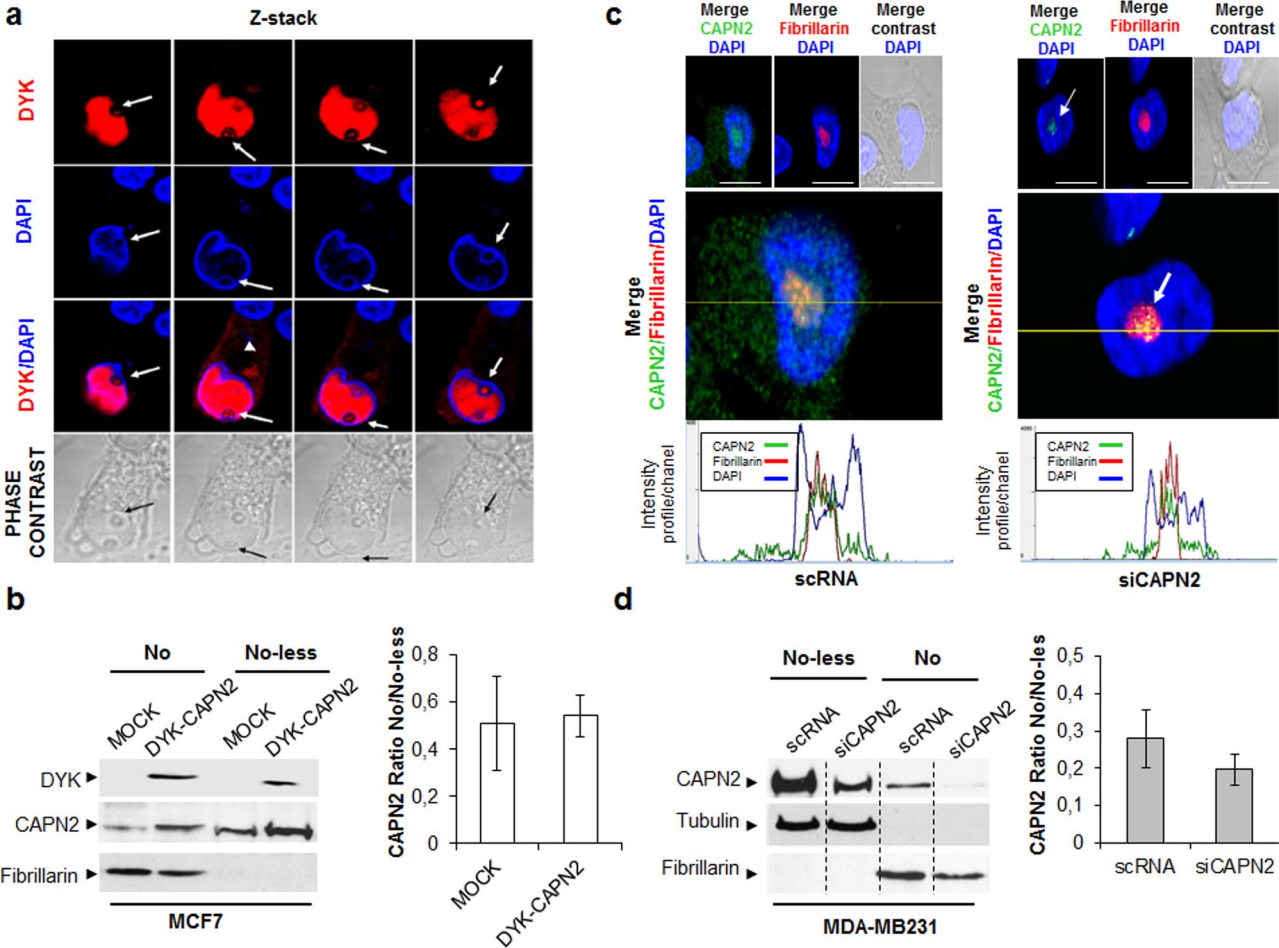
Specificity and expression-dependent localization of CAPN2 in nucleoli of BCCs. (**a**) Subnuclear distribution of ectopically expressed DYK-CAPN2 in MCF-7 cells analyzed by fluorescence staining with anti-DYK antibody (red) or DAPI (blue). Z-stack images (left to right) are shown. DYK-CAPN2 detected in nucleoli (arrow) and cytosol (arrowhead) is pointed out. Scale bars 20 μm. (**b**) Subnuclear distribution of DYK-CAPN2 analyzed by western blot in nucleolar (No) and nucleolar-less (No-less) fractions of MCF-7 cells transfected with either MSCV (Mock) or DYK-CAPN2 expression vector. Nucleolar CAPN2 was analyzed with both, anti-DYK and anti-CAPN2 antibodies. Fibrillarin was used to assess the purity of No fractions. The ratio of No/No-less CAPN2 is represented as mean ± SEM. No significant differences were found among both groups (n = 3). (**c**) Immunofluorescence analysis of CAPN2 in scRNA/siCAPN2 MDA-MB-231 transfected cells. Merge images of DAPI (blue) and either, CAPN2 (green), fibrillarin (red) or phase contrast are shown. Scale bars 20 μm. Representative images of all cells are shown. Magnification and H intensity profiles/channel exhibit the presence of residual CAPN2 (arrow) in nucleoli of CAPN2-silenced cells. (**d**) Subnuclear distribution of CAPN2 analyzed by western blot in No and No-less fractions of MDA-MB-231 cells transfected either with, scRNA or siCAPN2. Fibrillarin (No) and tubulin (No-less) were used to assess the purity of fractions. Data were quantified and plotted as mean CAPN2 No/No-less ratio ± SEM. No significant differences were found (n = 6). Juxtaposed images from different lanes in the same blot are separated by dotted lines in (**d**). Full-length blots are shown in Supplementary Fig. S14.

Epitope-tagged CAPN2 (DYK-CAPN2) was immunolocalized in both, the cytosolic and nuclear compartment of MCF-7 cells (a cell line with almost undetectable levels of endogenous nuclear CAPN2) (Fig. 2A). However, in order to analyze the subnuclear localization of ectopically over-expressed CAPN2, the high intensity of fluorescence signal was reduced. Thus, Flag-tagged CAPN2 was barely distinguished in the cytosol (arrowhead, Fig. 2A) and strongly detected in nuclei, where the protease was more concentrated.

As shown in Z-stack images and western blot with anti-DYK antibodies (Fig. 2A,B), ectopic DYK-CAPN2 accumulated into the nucleolus of transiently transfected cells, further confirming the specificity of CAPN2 nucleolar localization.

On the other hand, CAPN2 silencing in MDA-MB-231 cells (the cell line with the highest nuclear CAPN2 levels) strongly decreased the accumulation of CAPN2 in the whole cell (Fig. 2C,D). Although overall CAPN2 levels were efficiently down-regulated (70% decrease Supplementary Fig. S2) by siCAPN2, a 30% fraction of CAPN2 was still expressed. Accordingly, traces of nucleolar CAPN2 were detected in the nucleolus of CAPN2-silenced cells (Fig. 2C, arrow). Most likely nucleolar proteins concentrated in such a reduced area are more efficiently detected. Alternatively, as described for other proteins^23,24^, CAPN2 half-life could be higher in the nucleolus than in the rest of the cell.

Whatsoever, the effect of decreasing or increasing relative protein levels can be analyzed. We reasoned that the No/No-less ratio of overexpressed or downregulated CAPN2 would be constant in case the abundance was a key determinant for its nucleolar accumulation. CAPN2 No/No-less ratio was not significantly different when comparing mock and CAPN2-overexpressing MCF-7 cells (Fig. 2B) or scRNA and siCAPN2-transfected MDA-MB-231 cells (Fig. 2D). The higher levels of CAPN2, the higher accumulation into nuclei and nucleoli. On the whole, these data confirm the specificity of CAPN2 subnuclear distribution and, point out to CAPN2 abundance as an important determinant for its nucleolar localization in proliferating cells.

CAPN2 has no recognized nucleolar localization signal (NoLS). However, nucleolar proteins are not known to share a common NoLS. Nucleolar localization motifs are characterized by R/K rich sequences and positively charged residues, overlapping nuclear localization signals (NLS) and disorganized structures^25–27^. A bioinformatics-based analysis of CAPN2 (Supplementary Figs. S3–S6) predicted a non-consensus sequence fulfilling all the above mentioned conditions to be recognized as a NoLS.

Nevertheless, it seems that additional factors are needed to target proteins to the nucleolus. NoLS, rather than a nucleolar-recruiting signal, is thought to mediate the interaction of nucleolar proteins, rDNA or rRNA with those non-nucleolar proteins to be imported or retained into the nucleolus^28^. In that sense, we have previously described that CAPN2 binds to the core promoter of rDNA in colorectal cancer cells^13^. In addition to rDNA, CAPN2 might interact with nucleolar proteins, as also predicted in our bioinformatics analysis (Supplementary Fig. S7). Similarly, CAPN3 translocates to nucleoli by interaction with the nucleolar protein Def^29^. It seems that the membrane-less structure of nucleoli might also contribute to the internalization/retention of proteins.

The absence of membrane enables nucleoli to rapidly assemble or dissolve in response to growing stimuli. At metaphase, as the nucleolus disassembles many nucleolar proteins associate with other molecules in the nucleoplasm^20^. It is tempting to speculate that the high concentration of CAPN2 in nuclei of TNBC cells might favour its specific interaction with nucleolar proteins or rDNA. It seems reasonable to think that nucleoli assembly at the end of mitosis might retain higher amount of CAPN2 in CAPN2-overexpressing BCCs. Whether the nucleolar localization of CAPN2 involves its interaction with additional proteins remains to be explored. In the future, the identification of CAPN2 interacting proteins in nucleoli would be an important issue to completely unveil the molecular determinants of CAPN2 nucleolar localization.

### Identification of the CAPN2-dependent components of nucleoli

Accumulation of CAPN2 into the nucleolus could be crucial for the function/regulation of nuclear proteins. Nucleolar extracts from scRNA and siCAPN2 MDA-MB-231 transfected-cells were analyzed by 2D-DIGE. Nucleoli are membrane-less structures, continuously subjected to assembly/disassembly during cell cycle^20^. Therefore, nucleolar extracts from asynchronous cells are expected to include proteins from nucleoli and perinucleolar region.

**Figure 3:**
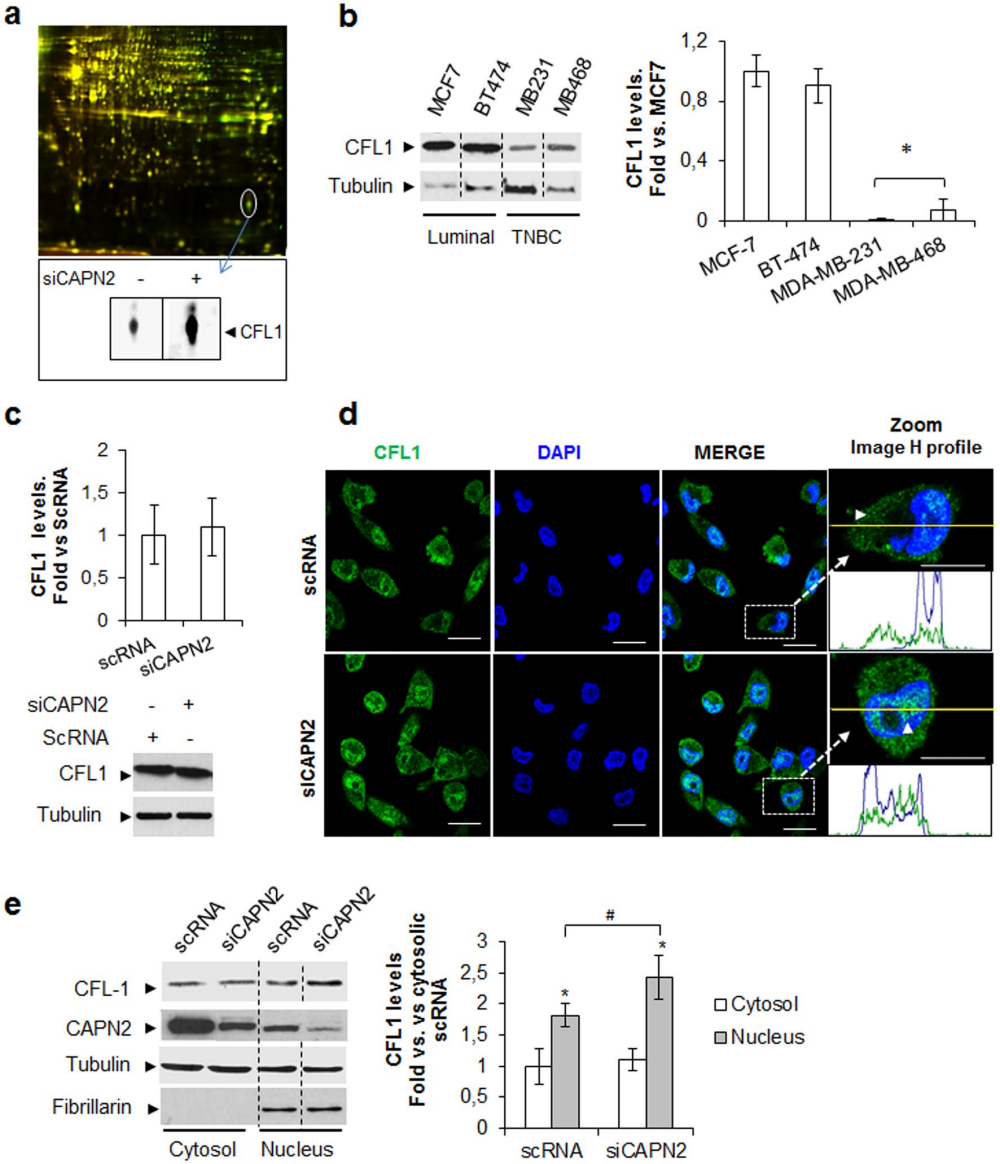
Identification of CFL1 as a protein with a CAPN2-dependent subcellular distribution. (**a**) 2D-DIGE of nucleolar extracts from MDA-MB-231 transfected with scRNA and siCAPN2. A representative gel is shown (n = 4). Inset shows unmerge images of the spot identified as CFL1. (**b**) Total CFL1 protein levels in BCC lines. Data (n ≥ 3) were quantified, normalized with tubulin and represented as mean fold ± SEM. *p ≤ 0.01 vs. MCF-7 cells. (**c**) Western blot of total CFL1 levels in scRNA/siCAPN2-transfected MDA-MB231 cells. Data (n = 6) were quantified, normalized with tubulin and represented as mean fold ± SEM vs. scRNA. (**d**) CFL1 (green) immunostaining and DAPI (blue) in MDA-MB-231 cells transfected with scRNA or siCAPN2. (n = 6). H intensity profiles/channel are shown. Scale bars 20 μm. (**e**) Subcellular distribution of CFL1 in cytosolic and nuclear fractions. Data (n ≥ 3) were quantified, normalized with fibrillarin (N) or tubulin (C), and plotted as mean fold ± SEM vs. scRNA cytosolic fractions. *p ≤ 0.01 vs scRNA cytosolic fractions. ^#^p ≤ 0.05 vs. scRNA nuclear fractions. Juxtaposed images from different lanes in the same blot are separated by dotted lines in (**b**, **e**). Full-length blots are presented in Supplementary Fig. S14.

A total of 11 differentially represented spots were excised from 2D-DIGE gels and identified by LC–MS/MS (Supplementary Table S1). The actin-severing protein cofilin-1 (CFL1) was found among the upregulated proteins in the nucleolus of CAPN2-depleted cells (Fig. 3A). CFL1 is required for actin cytoskeleton reorganization, and for a number of physiological and tumorigenic processes^30,31^.

Focusing on this CAPN2-target, CFL1 protein levels were analyzed in BCC cell lines. As shown in Fig. 3B, luminal cells have higher levels of CFL1 than TNBC cells. The opposite has been reported for CAPN2^8^. In agreement with this, low CFL1 levels detected in TNBC cells could be the result of CAPN2-mediated cleavage of CFL1. Strikingly, truncated CFL1 or differences in total CFL1 levels were not observed upon CAPN2 silencing in MDA-MB-231 cells (Fig. 3C).

Other nucleolar proteins either upregulated or downregulated in CAPN2-knocked-down cells were also analyzed, but no significant differences were found (Supplementary Fig. S8). Hence, the CAPN2-dependent composition of nucleoli and perinucleolar region does not seem to result from the proteolytic activity of CAPN2 on identified proteins, but rather on the subcellular distribution of nuclear components.

CAPN2-mediated distribution of CFL1 was studied in CAPN2 knocked-down MDA-MB-231 cells by immunofluorescence staining (Fig. 3D). CFL1 was distributed throughout the cytoplasm and nucleoplasm of scRNA control cells. Fluorescence intensity analysis revealed a stronger accumulation of CFL1 in the region extending from the cell membrane to ~ 1–2 μm into the cell surrounding a large region of low staining (zoom image asterisk and H profile). Although still detected into the cytoplasm, ~ 45% of CAPN2-depleted cells showed an important fraction of CFL1 accumulated at interchromatin spaces as nuclear speckles, at the perinucleolar region (arrowhead) and, although almost undetectable, into nucleoli. The effect of CAPN2 expression in nuclear CFL1 levels was confirmed by western blot in subcellular fractions of siCAPN2 cells (Fig. 3E). No change of CFL1 levels could be detected in the cytosolic compartment upon CAPN2 silencing. However, according to the CFL1 distribution in confocal images, a significant increase of nuclear CFL1 levels was observed in siCAPN2 compared to scRNA-transfected cells.

All in all, these data might suggest a role for CAPN2 in the modulation of nuclear rather than total, CFL1 levels.

### Role of CAPN2 in the control of phospho-cofilin-1 levels

**Figure 4:**
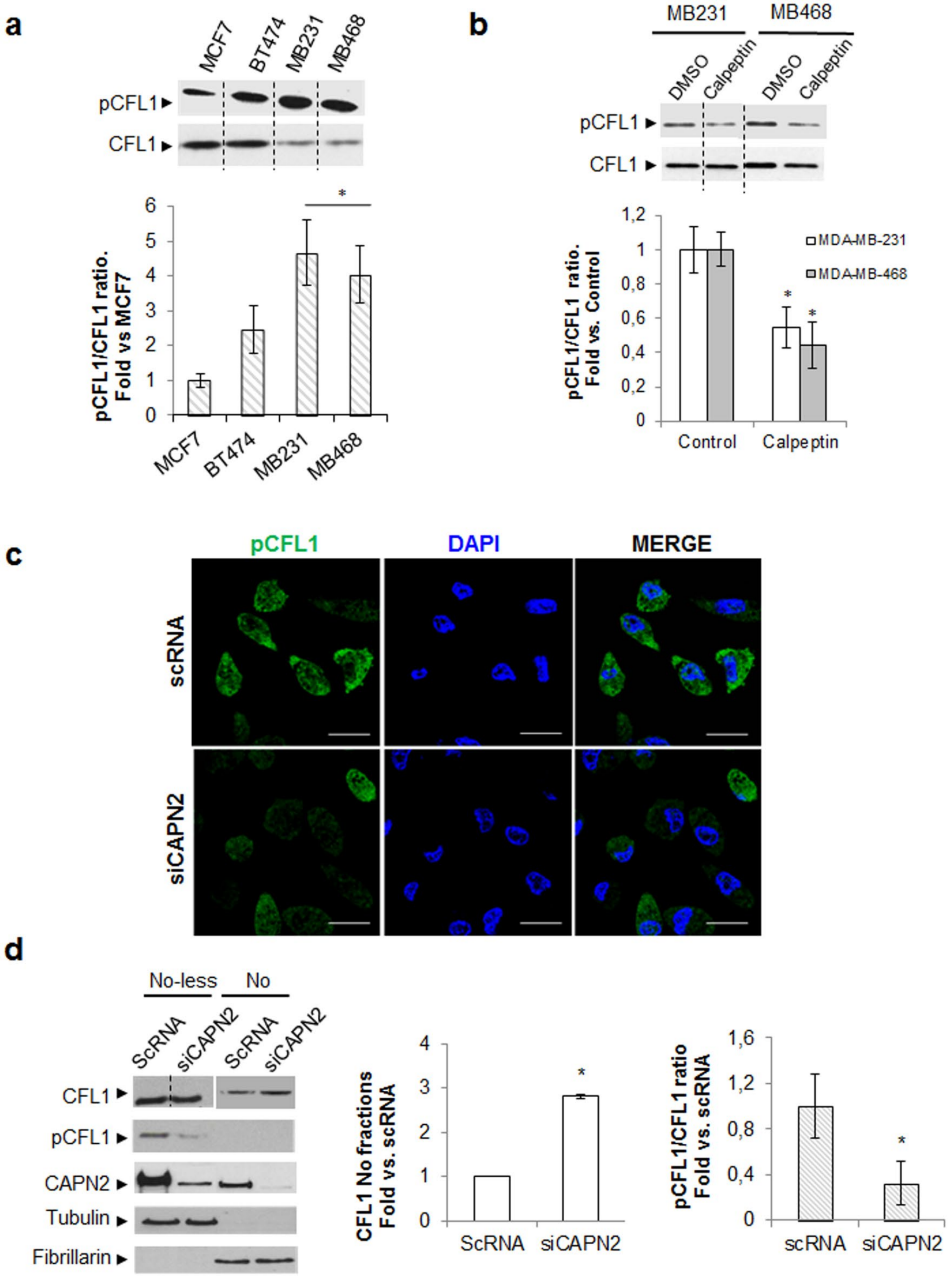
Effect of CAPN2 expression in Cofilin-1 phosphorylation rate and subnuclear distribution. (**a**) Immunoblots of pCFL1 and CFL1 in whole extracts from BCC cell lines. Data (n ≥ 3) were quantified and represented as the ratio pCFL1/CFL1 mean fold ± SEM. *p ≤ 0.01 vs. MCF7 cells. (**b**) Immunoblots of pCFL1 and CFL1 in whole protein extracts from TNBC cells (n = 3) cultured for 24 h in the presence of vehicle (DMSO) or 50 μM calpeptin. Data (n ≥ 3) were quantified and the ratio pCFL1/CFL1 represented as mean fold ± SEM vs. DMSO-treated cells. *p ≤ 0.01. (**c**) Immunofluorescence staining of pCFL1 (green) and DAPI (blue) in MDA-MB-231 cells transfected with scRNA/siCAPN2 (n = 6). Scale bars 20 μm. (**d**) Subnuclear distribution of CFL1 and pCFL1 analyzed by western blot in nucleolar (No) and nucleolar-less fractions (No-less) from MDA-MB-231 cells transfected with scRNA/siCAPN2. Data (n ≥ 3) were quantified, normalized with fibrillarin (No) or tubulin (No-less), and plotted as nucleolar CFL1 mean fold ± SEM vs. scRNA transfected-cells *p ≤ 0.01 or as the ratio pCFL1/CFL1 mean fold ± SEM. *p ≤ 0.05 vs. scRNA transfected-cells. Juxtaposed images from different lanes in the same blot are separated by dotted lines in (**a**, **b**, **d**). Full-length blots are presented in Supplementary Fig. S14.

Phosphorylation of CFL1 inhibits its actin-severing activity and promotes changes in its subcellular distribution^30^. Phospho-CFL1 to CFL1 (pCFL1/CFL1) ratio, critical for tumorigenesis^30–32^, was significantly higher in TNBC cells than in luminal cells (Fig. 4A). A positive correlation between CAPN2 protein levels and pCFL1/CFL1 ratio (r = 0.8159, p = 0.02) was found in the luminal and TNBC cell lines used in this study (Supplementary Fig. S9). Thus, we hypothesized that CAPN2 might be involved in the regulation of CFL1 phosphorylation pathway.

In agreement with this, calpeptin (a specific inhibitor of calpain activity) partially inhibited CFL1 phosphorylation in TNBC cell lines and consequently, the ratio pCFL1/CFL1 was significantly decreased when compared to untreated controls (Fig. 4B).

Distribution of pCFL1 was analyzed by confocal microscopy in control and CAPN2-depleted cells (Fig. 4C). As previously reported^33^, pCFL1 was clearly more abundant in the cytosolic compartment than in the cell nucleus. In agreement with results obtained with calpain inhibitor, pCFL1 levels also decreased in most of siCAPN2-transfected cells (~ 77%) analyzed by immunofluorescence staining (Fig. 4C) and in nucleolar-less fractions analyzed by western blot (Fig. 4D). These data further confirm that CAPN2 is, at least in part, involved in the phosphorylation pathway of CFL1.

Interestingly, although no traces of pCFL1 could be detected in nucleolar fractions, CFL1 levels increased upon CAPN2 silencing. These observations not only validate data obtained in our proteomic analysis, but also lead to hypothesize that CAPN2 might be involved in CFL1 distribution through the modulation of its phosphorylation pathway. Supporting this hypothesis, it has been reported that pCFL1 is mainly localized in the cytosol while its dephosphorylated form adopts a nuclear localization^33–35^. However, although out of the scope of this paper, important questions related to the mechanisms for CFL1 phosphorylation and distribution are yet to be determined*.*

### Analysis of cofilin-1 phosphorylation pathway as indirect target of CAPN2

CAPN2 could induce CFL1 phosphorylation by the indirect activation of its phosphorylation pathway. Since both, LIMK1 and LIMK2 are known to phosphorylate CFL1, we previously confirmed the prominent role of LIMK1 on CFL1 phosphorylation in MDA-MB-231 cells as reported^36^. A significant reduction in the pCFL1/CFL1 ratio was observed after LIMK1 silencing (Supplementary Fig. S10). We could not detect any CAPN2-dependent effect on slingshot/chronophin (SSH/CIN) phosphatase (data not shown) known to dephosphorylate CFL1^27^.

**Figure 5:**
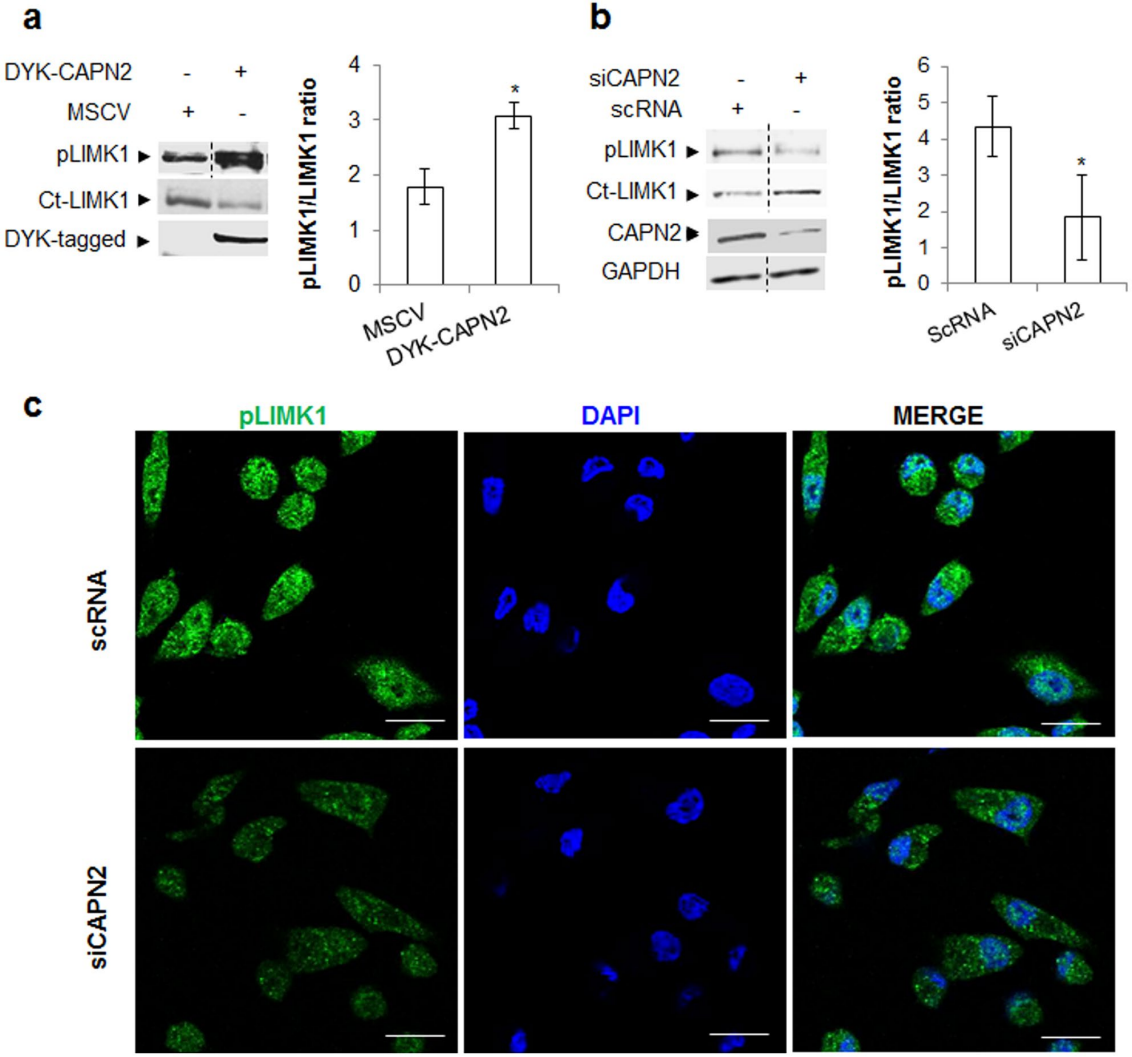
CAPN2 promotes phosphorylation of LIMK1 in BCCs. (**a**) p(Thr508)LIMK1 and total Ct-LIMK1 levels analyzed by western blot in MCF-7 cells transfected with MSCV empty vector or DYK-CAPN2. Anti-DYK antibody was used to assess transfection efficiency. Data (n ≥ 3) were quantified and plotted as mean pLIMK1/LIMK1 ratio ± SEM. *p ≤ 0.05 vs. MSCV. (**b**) p(Thr508)LIMK1 and total Ct-LIMK1 levels in MDA-MB-231 cells transfected with scRNA/siCAPN2. Anti-CAPN2 and anti-GAPDH antibodies were used to assess transfection efficiency and equal loading respectively. Data (n ≥ 3) were quantified, and plotted as mean pLIMK1/LIMK1 ratio ± SEM. *p ≤ 0.05 vs. scRNA transfected-cells. (**c**) p(Thr508)LIMK1 (green) distribution in MDA-MB-231 cells transfected with scRNA/siCAPN2 (n = 3) was analyzed by immunofluorescence staining. Nuclei stained with DAPI (blue) and merge images are shown. Scale bars 20 μm. Juxtaposed images from different lanes in the same blot are separated by dotted lines in (**a**, **b**). Full-length blots are presented in Supplementary Fig. S14.

Over-expression of DYK-CAPN2 in MCF-7 cells induced the phosphorylation of (Thr-508)-LIMK1 (Fig. 5A and Supplementary Fig. S10). Interestingly, ectopic expression of CAPN2 reduced levels of total LIMK1. Accordingly, pLIMK1/LIMK1 ratio was significantly increased in DYK-CAPN2-transfected cells. Conversely, pLIMK1/LIMK1 ratio was reduced upon CAPN2 silencing. (Fig. 5B). Most cells stained with p(Thr-508)-LIMK1 antibody showed an overall decrease of pLIMK1 levels after CAPN2 depletion (~ 90%), although no apparent change in a particular pattern of pLIMK1 distribution was detected (Fig. 5C).

In agreement with these data, CAPN2 has been reported to trigger the ROCK-RhoA-mediated phosphorylation of LIMK1 at Thr-508 during cell migration or synaptic plasticity^7^. However, other mechanisms for LIMK1 activation have been suggested^37^. Reported data show similar levels of pCFL1 after ectopic expression of nuclear-LIMK1 or cytosolic-LIMK1 in MDA-MB-231 cells, despite levels of p(Thr-508)-LIMK1 were much lower in the nucleus than in the cytosol^36^.

### Direct interaction of CAPN2 and LIMK1

**Figure 6:**
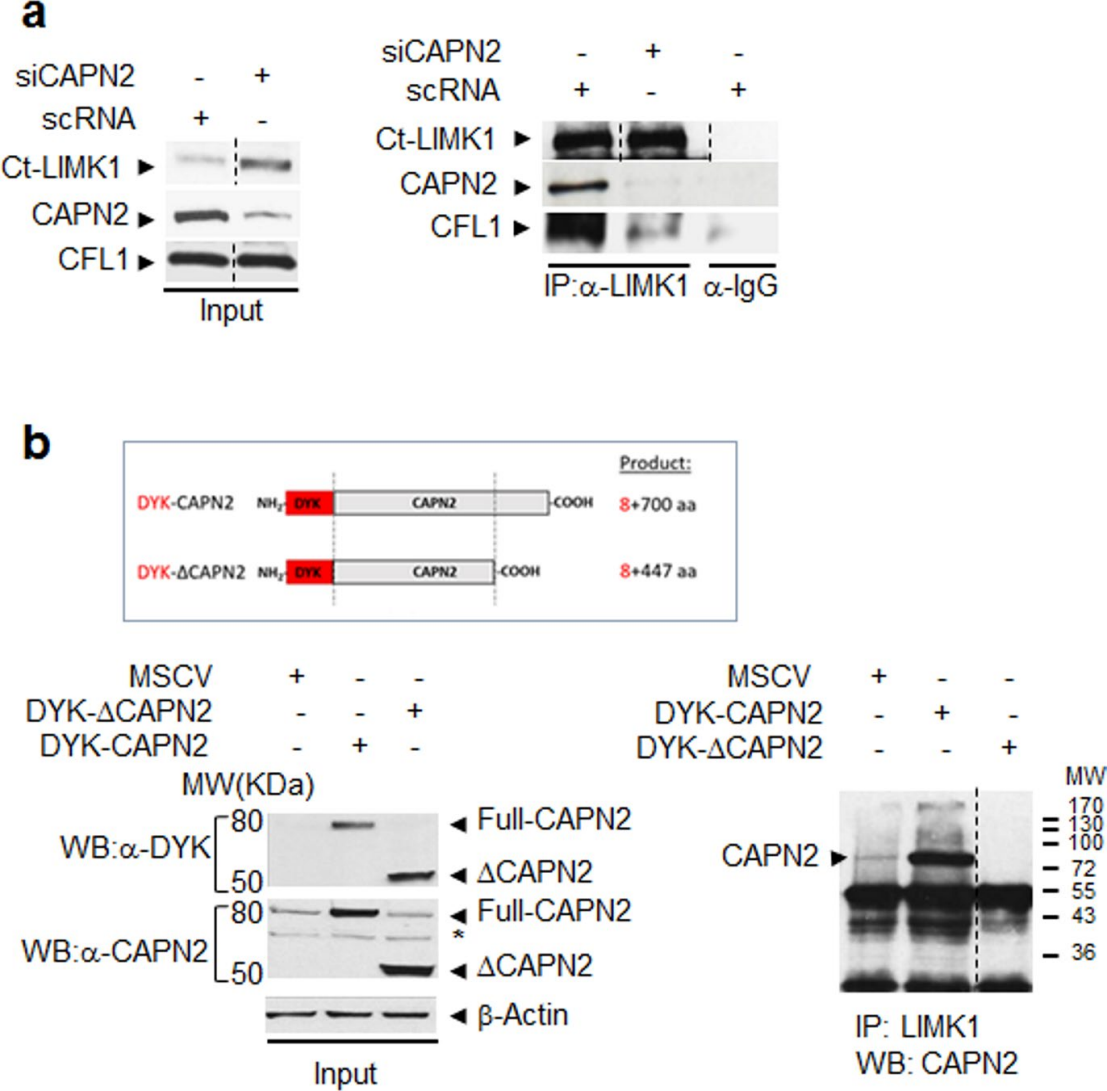
Direct interaction of CAPN2 and LIMK1 in MDA-MB-231 cells. (**a**) Coimmunoprecipitation of LIMK1 and CAPN2 in protein extracts from MDA-MB-231 cells transfected with scRNA/siCAPN2. Whole extracts were immunoprecipitated with anti-Ct-LIMK1 and analyzed by western blot with anti-CAPN2 and anti-CFL1 antibodies. Inputs are shown. (**b**) Full-length DYK-CAPN2 or DYK-∆CAPN2 was overexpressed in MCF-7 cells (graphical representation is shown). Protein extracts were immunoprecipitated with anti-Ct-LIMK1 and analyzed by western blot with anti-CAPN2 antibody. Uncropped image is shown. Inputs were analyzed with DYK, CAPN2 or β-actin antibodies (left panel). (*) Unspecific bands recognized by CAPN2 antibody. Juxtaposed images from different lanes in the same blot are separated by dotted lines. Full-length blots are presented in Supplementary Fig. S14.

Protein binding to LIM domains is known to induce a LIMK1 conformational change and unmask the kinase domain which can be subsequently phosphorylated and activated^37^. CAPN2/LIMK1 direct binding was analyzed by coimmunoprecipitation followed by western blot in total extracts of MDA-MB-231 cells transfected with either, scRNA or siCAPN2. CAPN2/LIMK1 coimmunoprecipitation was observed in control but not in CAPN2 knocked-down samples (Fig. 6A). Down-regulation of CAPN2 also prevented LIMK1 binding to its substrate CFL1, suggesting that CAPN2/LIMK1 interaction is a major determinant for LIMK1 activity on CFL1.

In addition to LIM domains, LIMK1 contains a PDZ domain known to be a protein–protein interaction module^37^. A type I PDZ binding domain has been recently identified at the C-terminus of CAPN2 (Supplementary Fig. S11)^38^. To assess the role of the C-terminus of CAPN2 in this interaction, MCF-7 cells were transfected with a DYK-tagged expression vector coding for either, full length or truncated CAPN2 (∆CAPN2) which retains the catalytic domains but lacks its C-terminal portion (Fig. 6B). Since the PDZ domain of CAPN2 was at its C-terminus, an N-terminal epitope-tagged expression vector was selected.

LIMK1 coimmunoprecipitated with full-length DYK-CAPN2, but failed to coimmunoprecipitate with DYK-∆CAPN2 (Fig. 6B). It could be argued that since C-terminally ΔCAPN2 lacks the PEF domain it will not dimerize with the CAPNS1 regulatory subunit and consequently, ΔCAPN2 could be unstable or even mislocalized relative to full length CAPN2. Truncated CAPN2 was over-expressed for 48 h in our experiments, time enough to compromise the levels of an unstable protein. Nevertheless, our analysis by western blot showed even higher ΔCAPN2 protein levels than epitope-tagged full-length CAPN2 (Fig. 6B, inputs) and no signs of protein degradation. In addition, subcellular localization of ΔCAPN2 was analyzed by western blot and confocal microscopy in MCF-7 cells (Supplementary Fig. S12). Truncated CAPN2 exhibits the same localization in nuclei and nucleoli as full-length CAPN2. Therefore, mislocalization or instability of ∆CAPN2 was not preventing the interaction between LIMK1 and ΔCAPN2 in our experiments.

These data not only demonstrate the specificity of CAPN2/LIMK1 interaction, but also support the direct interaction of LIMK1 with the C-terminal domain of CAPN2.

### LIMK1 as a target of CAPN2 cleavage-activity

**Figure 7:**
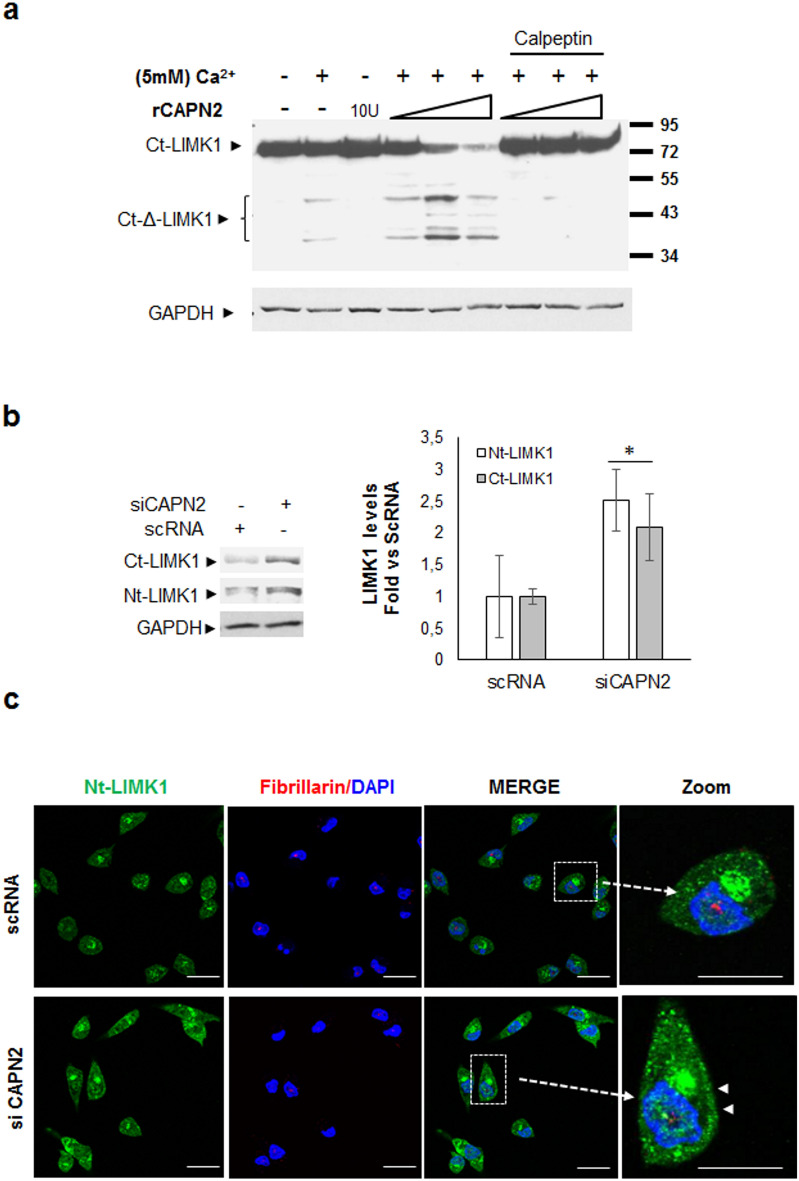
CAPN2-mediated cleavage of LIMK1. (**a**) In vitro CAPN2-cleavage assay. Protein extracts from MDA-MB-231 cells incubated with increasing concentrations (1, 5 and 10U) of recombinant CAPN2 in the presence or absence of Ca^2+^ and 50 µM calpeptin. Truncated (∆LIMK1) and full length LIMK1 were analyzed by western blot with Ct-LIMK1 antibody. GAPDH was used to show cleavage specificity. A representative uncropped image is shown. (**b**) Ct-LIMK1 and Nt-LIMK1 levels in MDA-MB-231 cells transfected with scRNA/siCAPN2. LIMK1 protein levels were quantified, normalized with GAPDH and plotted as mean (n ≥ 3) fold ± SEM. *p ≤ 0.05 vs. scRNA transfected-cells. (**c**) Confocal images of Nt-LIMK1 (green), Fibrillarin (red) and DAPI (blue) in MDA-MB-231 cells transfected with scRNA/siCAPN2. Magnification shows a merge image of a representative cell. Scale bars 20 μm. Full-length blots are shown in Supplementary Fig. S14.

Cleavage of N-terminal autoinhibitory domain of LIMK1 has been described as another mechanism for LIMK1 activation^37^. LIMK1 has several predicted sites for CAPN2 cleavage (Supplementary Fig. S11). The identification of LIMK1 as a CAPN2-substrate was analyzed in a cell-free assay with recombinant CAPN2 (rCAPN2) followed by western blot (Fig. 7A). Full-length LIMK1 was proteolyzed in a dose-dependent manner by rCAPN2 in the presence of Ca^2+^. Truncated fragments of LIMK1 strongly increased in the presence of 1U and 5U of recombinant CAPN2 and Ca^2+^ when compared to control samples. Moreover, LIMK1 fragments are observed even in the sole presence of Ca^2+^, most likely resulting from endogenous CAPN2 activity (lane 2, Fig. 7A). As expected, a high non-physiological CAPN2 concentration (10U) produces extensive proteolysis of full length LIMK1. Truncated and surely more unstable forms of LIMK1 are reduced under such CAPN2 concentration. Fragments of other reported calpain-substrates are also reduced when incubated with high concentrations of rCAPN2^8,9^. Since GAPDH protein levels were not affected, the unspecific proteolysis of LIMK1 by rCAPN2 was discarded.

Interestingly, two major LIMK1 cleavage fragments (~ 38–50 KD) were detected. Caspase-3-mediated cleavage of LIMK1 also results in ~ 45–60 KD LIMK1 fragments which retain CFL1 phosphorylation activity^39^. In the presence of calpeptin, full-length LIMK1 was not affected by rCAPN2 and truncated LIMK1 was not detected.

These data show the specificity of CAPN2-mediated cleavage of LIMK1. Conversely, a significant increase of full-length LIMK1 was detected upon CAPN2 down-regulation in MDA-MB-231 cells by western blot (Fig. 7B) and immunofluorescence staining (Fig. 7C). Interestingly, while an overall increase of LIMK1 levels was observed upon CAPN2 depletion, a high percentage of cells (53%) also showed LIMK1 accumulation at the cell periphery (arrowhead).

Unfortunately, truncated LIMK1 could not be detected in these experiments. The antibody against Ct-LIMK1 detected several lower molecular weight proteins when films were overexposed. Most probably, the amount of ∆LIMK1 was at the detection limit in total protein extracts making very difficult to distinguish between unspecific or specific recognition by the antibody.

Samples from MSCV and DYK-CAPN2 MCF-7-transfected cells were electroblotted and membranes excised and incubated with Ct-LIMK1 antibody. Since MCF-7 cells express low levels of LIMK1, the lower part of membranes was overexposed to detect ∆LIMK1 (Supplementary Fig. S10). At least one band, also detected in MSCV-control cells, seemed to specifically increase upon CAPN2 overexpression. Protein extracts from siLIMK1-transfected cells were analyzed by western blot with the same anti-Ct-LIMK1 antibody. A low molecular weight protein (~ 43–45 KD) observed in control cells, was not detected in siLIMK1 transfected cells (Supplementary Fig. S10). Conversely, other unspecific bands detected by the antibody were not affected by LIMK1 silencing. These data suggest that most likely the increased bands recognized by the antibody in DYK-CAPN2 samples could be indeed ∆LIMK1. Interestingly, when analyzing a more restrictive fraction of LIMK1, such as p(T508)LIMK1, the same ~ 43–45 KD band was detected.

Although it is still possible that other kinases from the ROCK/RhoA pathway have a synergistic role in LIMK1 activation^40^, these data suggest a CAPN2-mediated cleavage of LIMK1 as a novel mechanism for LIMK1 activation. In addition, it could be speculated that CAPN2-mediated cleavage of LIMK1 could be part of its activation mechanism or alternatively, it could also be involved in the subcellular distribution of LIMK1. However, this hypothesis should be further confirmed.

LIMK1 is known to be localized in nuclear speckles (www.proteinatlas.org/ENSG00000106683-LIMK1/cell#human). Most proteins from nuclear speckles can also be found at other nuclear locations, although their specific functions, post-translational modifications and interacting partners need to be elucidated^41^. Proteins from nuclear speckles and nucleoli continuously dissociate from and associate to their respective sub-compartments and the rate of these movements has been shown to be dependent on the transcription activity and the phase of the cell cycle. It has been suggested that nuclear proteins might roam the nucleus in search of specific binding partners^42^. This highly dynamic context would help CAPN2/LIMK1 interaction. Cleavage or mutation of low complexity regions in proteins from nuclear speckles is known to alter protein–protein, protein–RNA interactions and localization to nuclear speckles^43^. The differential subcellular distribution of LIMK1 upon CAPN2-silencing might reflect a change in its activity or interaction with its partner CFL1.

### Effect of CAPN2 expression on CFL1 phosphorylation during cell cycle

Phosphorylation and redistribution of CFL1 during mitosis follows the same pattern as LIMK1 activation at prometaphase^44,45^, but not that of ROCK/RhoA which peaks at telophase^46^. Actually, mitotic activation of LIMK1 has been reported to be driven by other mechanisms than phosphorylation at Thr-508 by the ROCK/RhoA pathway^44^.

The role of CAPN2 on mitotic activation of LIMK1 and subsequent phosphorylation of its substrate CFL1 was studied during cell cycle in asynchronous cells in a context not disturbed by phase-specific inhibitors. For instance, nocodazol commonly used to block cell cycle at prometaphase, is known to alter spindle positioning^47,48^, an important LIMK1-regulated event^45^. Cells transfected with either, scRNA or siCAPN2 were stained with CFL1 or pCFL1 antibodies and cell cycle progression monitored by DAPI staining and cell morphology.

**Figure 8:**
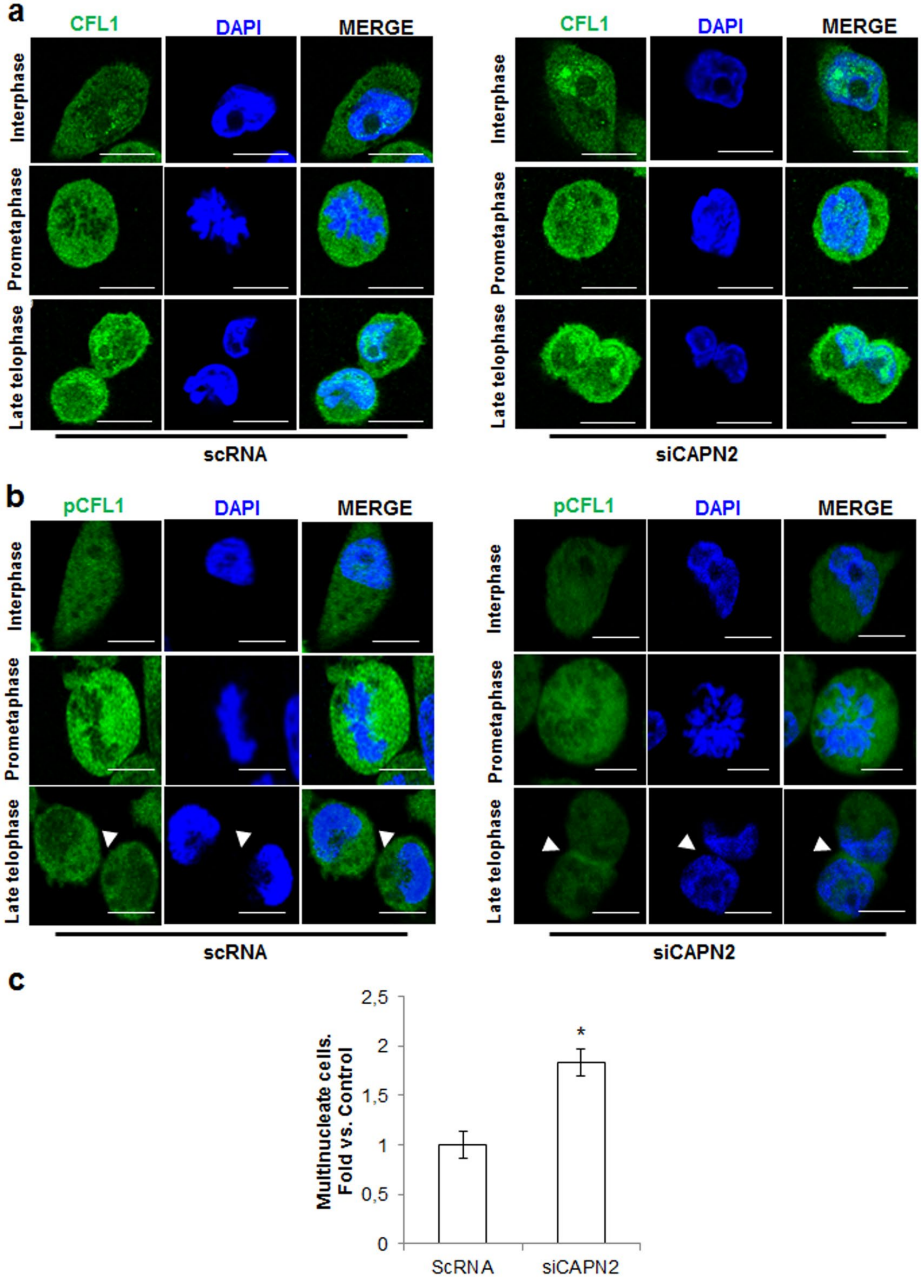
Effect of CAPN2 expression on CFL1 distribution and phosphorylation during cell cycle. Immunofluorescence staining of (**a**) CFL1 (green) and (**b**) pCFL1 (green) in asynchronous MDA-MB-231 cells transfected with scRNA/siCAPN2. Scale bars 20 μm. Representative images of almost all cells found at prometaphase and late telophase in scRNA or siCAPN2-transfected cells are shown. (**c**) Quantification of multinucleate cells in MDA-MB-231 transfected with scRNA or siCAPN2 and analyzed by flow cytometry. Values were plotted as mean (n = 4) fold ± SEM vs. scRNA transfected-cells *p ≤ 0.0001.

As previously reported for control cells^44,45^, at prometaphase the pCFL1/CFL1 ratio was strongly increased compared to neighbouring cells and pCFL1 homogeneously distributed surrounding chromosomes (Fig. 8A,B, left panels). At late telophase and interphase, the pCFL1/CFL1 ratio decreased at cell membranes and within the nucleus: dephosphorylated CFL1 accumulated into the nucleus and at the cell membranes, while pCFL1 translocated from the cell periphery into the cytoplasm, (Fig. 8B, left panel arrowhead).

In CAPN2-depleted cells, the pCFL1/CFL1 ratio was dramatically downregulated at any phase when compared to control cells (Fig. 8A,B, right panels). A modest increase of pCFL1 was observed at prometaphase compared to surrounding cells. At late telophase, pCFL1 translocation from the cell periphery to the cytoplasm was delayed (Fig. 8B, arrowhead). In addition, dephosphorylated CFL1 was abnormally accumulated into the cell nucleus at late telophase and interphase.

These data suggest that CAPN2 might be involved in the modulation of CFL1 phosphorylation and localization during cell cycle. Altered pattern of CFL1 phosphorylation leads to abnormal centrosome migration, delayed anaphase/telophase, cytokinesis fail and cell multinucleation^44,45,49^. Thus, multinucleation was analyzed in scRNA and siCAPN2-transfected cells. The number of multinucleate cells after CAPN2-downregulation was two-fold higher than in control cells (Fig. 8C and Supplementary Fig. S13). These data suggest that CAPN2 plays a crucial role during mitosis progression and cytokinesis through the modulation of CFL1 phosphorylation at critical steps of mitosis. In fact, we observed a higher percentage of cells at G2/M in siCAPN2-transfected cells than in scRNA controls (11 ± 0.53% vs. 7.27 ± 0.42%, p ≤ 0.0001). In agreement with this, CAPN2 depletion in C2.7 murine myoblast cell line or in HeLa cells resulted in defective chromosome alignment during prometaphase, unbalanced chromosome segregation during anaphase and aberrant mitosis^17,18^. However, in the before mentioned studies no mechanism for the effect of CAPN2 on cell mitosis was provided.

We could speculate that CAPN2 would be retained in the nucleolus of BCCs at interphase modulating rRNA biogenesis as we previously reported in colorectal cancer cells^13^, and released during mitosis to cleave and activate LIMK1 known to be involved in spindle positioning at prometaphase^45^. The same behavior has been observed in other proteins with eventual or typical nucleolar localization such as, Ki-67, nucleophosmin (B23) or fibrillarin^50–52^. These nucleolar proteins are retained in the nucleolus at interphase to modulate rRNA biogenesis and, released later for mitotic congression. Moreover, depletion of B23 or fibrillarin in HeLa cells also results in aberrant mitosis and abnormal nuclear morphology or cell multinucleation in asynchronous interphase cells^51,52^.

It has been recently shown that spindle positioning requires not only microtubule polymerization, but also actin polymerization at prometaphase^53^. CAPN2-mediated activation of LIMK1 would increase pCFL1 levels at prometaphase, promoting F-actin polymerization for the correct mitotic congression.

In addition to its function on nucleoskeleton remodeling, nuclear roles for CFL1 include the maintenance of nuclear structure, association with the three RNA polymerases and movement of chromosomes^30,31^. Consequently, our findings suggest a new mechanism for CAPN2 functions in the nuclear compartment involving the time-course of LIMK1 activation and CFL1 phosphorylation during cell cycle.

## Conclusions

The altered interaction between nuclear components leads to nuclear dysfunction and often to cancer development^20^. Our experiments indicate that differential CAPN2 expression according to the breast cancer subtype is an important determinant for its nucleolar localization in tumor cells. We show that the composition and presumably, the interaction of nucleolar proteins found in control cells change upon CAPN2 down-regulation. In addition, our data indicate that the modification of these interactions has profound effects in the cell. CAPN2-depletion results in altered mitosis and cell multinucleation. Data presented herein strongly suggest that CAPN2 plays a critical role in LIMK1-mediated phosphorylation of CFL1 which, although more evident during mitosis, also occurs at interphase. We propose a CAPN2-mediated cleavage and activation of LIMK1 as a novel mechanism to induce phosphorylation of CFL1.

In the future, unveiling the dynamics of nucleolar CAPN2 will be important to understand the complex network of nuclear interactions. Nuclear actin dynamics, modulated by pCFL1/CFL1 ratio, are involved in chromatin remodeling, transcription, RNA processing, intranuclear transport, nuclear export, and maintenance of the nuclear architecture^23,24^. Therefore, the role of CAPN2 in the nuclear compartment might be extended to an important number of actin-associated biological and pathological processes that need to be further investigated. Finally, high expression of CAPN2 and aberrant activation of the LIMK1/CFL1 axis found in breast cancer have been positively correlated with cancer development and metastasis^6,8,30,33^. A number of inhibitors of LIMK1/CFL1 expression and/or activation have been explored for their possible use in anticancer therapies^30,35^. CAPN2 might be an important target with therapeutic potential for the design of inhibitors of the LIMK1/CFL1 signaling pathway in breast cancer.

## Methods

### Materials

Primary and secondary antibodies used in western blots, immunoprecipitations and immunofluorescence staining are provided in Supplementary Table S2. Recombinant CAPN2 (208718) and calpain inhibitor Calpeptin (03-34-0051), were from Calbiochem.

### Cell culture

Human breast cancer cell lines were purchased from American Type Culture Collection (ATCC) including certificate of analysis and mutation sequencing data. Cell lines from either luminal, basal, or claudin-low subtypes were used in this study as representative models of breast cancer cells with different CAPN2 abundance^8^. Selected luminal cell lines include: MCF-7 (ATCC HTB-22) and BT-474 (ATCC HTB-20), and triple-negative cell lines were MDA-MB-468 (ATCC HT-132) and MDA-MB-231 (ATCC HT-26). Cells were cultured in DMEM Medium (Gibco) supplemented with 10% FBS, 1% penicillin/streptomycin (K952, Amresco) and l-glutamine (G7513, Sigma) under standard conditions. Only low-passage cell lines were used for the studies. For experiments cells were plated at a density of 12–13.10^4^/cm^2^ (MCF-7), 14–16.10^4^/cm^2^ (BT474) and 16–18.10^3^/cm^2^ (MDA-MB-231 and MDA-MB-468) and maintained in asynchronous culture under standard conditions for 48 h.

### CAPN2 overexpression

Expression plasmids coding for DYK-tagged full-length CAPN2 and DYK-tagged-∆CAPN2 (1–447) were commercially available from GenScrip. The cDNA of full length CAPN2 (0Hu23853C) and truncated CAPN2 (ΔCAPN2) (0Hu56639C) were cloned on pcDNA3.1(+)-N-DYK construct. DYKDDDDK-epitope was tagged to the N-terminus of both, full-length and truncated CAPN2.

MCF-7 cells were cultured under standard conditions until preconfluence (48 h after seeding). Pre-confluent MCF-7 cells were transfected using Lipofectamine 3000 (L3000008, Life Technologies) following manufacturer’s instructions. Transfection reagents were diluted in Opti-MEM (31985070, Gibco). CAPN2 and ΔCAPN2 transfection efficiency was analyzed by western blot at 48 h after transfection.

### CAPN2 and LIMK1 knockdown by esiRNA

Cells were transiently transfected either with CAPN2 (EHU025391-50UG), LIMK1 (EHU073301) or Universal Negative Control #1 siRNA (SIC001), all purchased from Sigma. MDA-MD-231 cells were transfected following forward-transfection protocol and Lipofectamine 3000 as Transfection reagent. Transfection reactions were carried out for 24 h. Dilutions of esiRNA and Lipofectamine were performed in Opti-MEM following manufacturer’s instructions. esiRNA transfection efficiency was analyzed either by RT-qPCR at 48 h and 72 h after transfection or by western blot at 72 h after transfection.

### Subcellular fractionation

#### Isolation of nuclear/cytosolic fractions

Nuclear and cytoplasmic fractions were isolated using the Nuclear Extract kit (Active Motif, 40010) according to manufacturer’s instructions. In brief, washed cells were pelleted and lysed in hypotonic buffer to obtain cytoplasmic fractions. Nuclear pellets were incubated in lysis buffer for 30 min with gentle agitation at 4 °C. Samples were then centrifuged and supernatants recovered as nuclear fractions.

#### Nucleoli isolation

Cells (3 × 10^6^) were seeded on T225 cm^2^ flasks and cultured as indicated above. Nucleoli isolation was performed following a slightly modified published protocol ^13^. Briefly, cells were washed 3 times and suspended in 1.5 ml cold Solution I (0.5 M sucrose, 3 mM MgCl_2_, protease and phosphatase inhibitors). Cells were sonicated (Sonics, VCX130) on ice (40% amplitude, 10 pulses 10 s on/off) and > 90% efficiency ensured under the microscope. Cell lysates were laid on the same volume of Solution II (1 M sucrose, 3 mM MgCl_2_, protease and phosphatase inhibitors) and 1800×*g* centrifuged (10 min, 4 °C). Supernatants were 1800×*g* centrifuged again (10 min, 4 °C) and collected as *nucleolar-less* fractions containing the whole cell extract except nucleoli. Pellets were suspended in RIPA solution containing protease and phosphatase inhibitors, sonicated (40% amplitude, 4 pulses of 10 s/50 s on/off) and 14,000×*g* centrifuged (10 min, 4 °C). Supernatants were collected as *nucleolar* fractions and stored at − 80 °C.

### Protein extraction and immunoblotting

Total protein was extracted in RIPA buffer supplemented with protease and phosphatase inhibitors. Equal amounts of protein were size-fractionated by SDS-PAGE gel electrophoresis and electroblotted onto nitrocellulose membranes (Protran, Whatman). When samples from different cell lines were to be compared, although run in different gels, these were electroblotted onto the same membrane and incubated with the specific primary and HRP-conjugated secondary antibody. Blots were developed by enhanced chemiluminescence reaction (ECL Detection Kit, GE Healthcare). Equal loading or fraction purity was confirmed by reprobing blots with α-tubulin, Nup98, GAPDH or fibrillarin antibodies. To avoid membrane stripping whenever possible, blots were cut and each strip incubated with different antibodies to detect several proteins in the same membrane as described in supplementary information (uncropped images).

### In vitro CAPN2 cleavage assay

Whole protein extracts from MDA-MB-231 cells were incubated for 20 min at 37 °C with different units of recombinant CAPN2 in the presence of 5 mM Ca^2+^. To confirm calpain-mediated cleavage of LIMK1, reactions were carried out in the presence or absence of the chemical inhibitor of calpain activity, calpeptin (50 μM). Reactions were stopped by dissolving samples in SDS/Laemmli buffer and finally LIMK1 cleavage was analyzed by Western blotting as described above.

### Immunoprecipitation

Proteins were extracted in RIPA buffer supplemented with phosphatase and protease inhibitors as described elsewhere. Dynabeads™ protein G (Invitrogen 10003D) were 10 min pre-incubated with α-LIMK1 (1:50) or Normal serum IgG antibodies in RIPA. Magnetic beads were washed three times in lysis buffer and further incubated with 200 μg of protein extracts. After four washes, pull-down proteins were eluted in 50 mM glycine, pH 2.8 and boiled in Laemmli buffer 5× for immunoblot analysis.

### Immunofluorescence analysis

Cells were cultured onto 13 mm Ø borosillicate Cover Glass (VWR 631-0149) and immunostained as described previously^10^. Briefly, cells were incubated with the indicated primary antibodies overnight at 4 °C, followed by the secondary antibody. To ensure the signal specificity a negative control (where cells were incubated with secondary but not primary antibody) was routinely included. Nuclei were counterstained with DAPI (Invitrogen). Pictures were acquired on a FV1000 Olympus confocal microscope. Fields with too few or too many cells were excluded. At least 4 fields for each independent experiment were analyzed. The average number of cells/field was usually 60 ± 10. Except for studies in single cells, the most representative image of all fields was always selected and shown as a section of ~ 10 cells.

### Analysis of multinucleate cells

Multinucleate MDA-MB-231 cells were analyzed by flow cytometry at the Cytometry Unit from Unidad Central de Investigación de Medicina (UCIM), Universitat de Valencia. Briefly, cells were trypsinized at 72 h after transfection with either scRNA or siCAPN2, fixed with 70% ethanol, stained with propidium iodide (PI/RNAsa from Immunostep) and run in a flow cytometer (BD FACS Vosse) using a FACSuite software. Subsequent analysis with ModFit LT (V4.1.7) software was used for multinucleate and cell cycle examination. In addition, cells cultured in coverslips were fixed and nuclei stained with DAPI. Phase contrast/DAPI merge images obtained with a FV1000 Olympus confocal microscope were used to see multinucleate cells.

### 2D-DIGE proteomic analysis

2D-DIGE analysis was performed in nucleolar extracts from control and CAPN2 knocked-down MDA-MB-231 cells (n = 4). Nucleolar samples from scRNA and siCAPN2-transfected cells were alternatively labelled with Cy-3 or Cy-5 to decrease inter-gel variability. Recombined samples from silenced and control cells were labelled with Cy-2 for normalization and separated on 24 cm Immobiline DryStrips (pH 3–11) and 12.5%-acrylamide SDS-PAGE gels. Gels were scanned with Typhoon 9400 Variable Mode Imager and image analysis performed using DeCyder software (v7.0, Amersham Biosciences, GE Healthcare) and SameSpots v5.0.1.0. Significance of statistically different proteins was calculated using Student’s t-test and accepted when the value was p ≤ 0.05. Spots from 11 differentially represented proteins were excised and identified by LC–MS/MS at the proteomics facility (SCSIE) at the University of Valencia.

### Bioinformatics-based predictions

Prediction of NoLS motifs, positively charged, NLS and NoLS overlapping, and unfolded regions as well as protein–protein interaction sites in CAPN2 (NP_001739) were analyzed by different predictions platforms as indicated in Supplementary Figs. S3–S7.

### Statistics

Data are presented as mean fold ± S.E.M. Statistical significance was estimated by one-sample Student’s t-test with SPSS software. Differences were considered significant at least at p ≤ 0.05. Spearman’s correlation rho test was used for correlation analysis where a Rs value from 0.8 to 1 indicated a strong correlation between the pair of groups tested. Independent experiments were conducted with a minimum of three replicates per condition to allow statistical comparison.

## Supplementary Information

Supplementary Information.

## Data Availability

All data generated or analyzed during this study are included in this published article and its online supplementary material file.
