# Supplementary material for: Cleavage and activation of LIM kinase 1 as a novel mechanism for calpain 2-mediated regulation of nuclear dynamics
Source: Sci Rep. 2021 Aug 11;11:16339. doi: 10.1038/s41598-021-95797-8 (PMC8358030; doi:10.1038/s41598-021-95797-8)
Supplement: Supplementary file 1 — Supplementary Information. [file 41598_2021_95797_MOESM1_ESM.docx]

**Cleavage and activation of LIM kinase 1 as a novel mechanism for Calpain 2-mediated regulation of nuclear dynamics.**

Rodríguez-Fernández L.*^1^, Company S. ^*1^, Zaragozá R. ^2, 3^, Viña J.R. ^1,2^ * & García-Trevijano E.R. ^1,2^

^1^ Departamento de Bioquímica y Biología Molecular. Facultad de Medicina. Universidad de Valencia. Spain

^2^ Fundación Investigación Hospital Clínico-INCLIVA. Valencia. Spain

^3^ Departamento de Anatomía y Embriología Humana. Facultad de Medicina. Universidad de Valencia. Spain

*Authors contributed equally to this work

Corresponding author: E.R. García-Trevijano

E-mail address: [elena.ruiz@uv.es](mailto:elena.ruiz@uv.es)

| **PROTEIN** | **ACCESSION NUMBER** | **SYMBOL** | **siCAPN2** |
| --- | --- | --- | --- |
| Apolipoprotein A-I **(*)** | NP_001304947 | APOA1 | Down |
| Neutrophil defensin 3 | NP_005208 | DEFA3 | Down |
| HLA class I histocompatibility antigen, Cw-6 alpha chain | NP_002108 | HLA-C | Down |
| Prelamin-A/C **(*)** | NP_733821 | LMNA | Down |
| Peptidyl-prolyl cis-trans isomerase A **(*)** | NP_066953 | PPIA | Up |
| V-type proton ATPase subunit B, brain isoform | NP_001684 | ATP6V1 | Up |
| Aldehyde dehydrogenase X, mitochondrial | NP_000683 | ALDH1B | Down |
| Phosphoglycerate mutase 1 | NP_002620 | PGAM1 | Up |
| Stathmin | NP_001177695.1  NP_981946.1 | STMN1 | Up |
| Nucleoside diphosphate kinase B | NP_002503 | NME2 | Up |
| Cofilin-1 | **NP_005498** | CFL1 | **Up** |

**Supplementary Table S1. Proteins differentially represented in nucleoli from MDA-MB-231 analyzed by 2D-DIGE.**  Nuclear extracts from MDA-MB-231 cells transfected with scRNA/siCAPN2 were analyzed by 2D-DIGE and differentially represented spots identified.11 differentially represented proteins with statistical significance (p≤0.05) are shown. Last column shows Increase/decrease of the indicated protein in nucleoli of siCAPN2 vs. control scRNA cells. (*) Several spots for the same protein were identified.

| **Primary Antibody** | **Analysis** | **Reference** |
| --- | --- | --- |
| Calpain-1 | WB/IF | #ab39170 (Abcam) |
| Calpain-1 | IF | #ab3589) (Abcam) |
| Calpain-2 | WB/IF | #2539 (Cell Signaling) |
| αTubulin | WB | #52866 (Abcam) |
| GAPDH | WB | #8245 (Abcam) |
| Fibrillarin | WB/IF | #NB300-269 (Novus Biologicals) |
| DYKDDDDK-tag | WB/IF | #A00187 (Genscript) |
| Cofilin-1 | WB/IF | #5175 (Cell Signaling) |
| Phospho (S3)-Cofilin-1 | WB/IF | #3313 (Cell Signaling) |
| Ct-LIMK-1 | WB/IF/IP | #3842 (Cell Signaling) |
| Nt-LIMK-1 | WB/IF | #PA5-14938 (Invitrogen) |
| Phospho-(T508)-LIMK-1 | WB/IF | #PA5-37629 (Invitrogen) |
| Nup98 | WB | # Mab6758 (Abnova) |
| β-Actin | WB | #8227 (Abcam) |
| **Secondary Antibody** | **Analysis** | **Reference** |
| Alexa fluor 488 anti-rabbit IgG | IF | #A11008 (Invitrogen) |
| Alexa fluor 594 anti-Mouse IgG | IF | #A11005 (Invitrogen) |
| Goat Normal serum | WB/IP | #X0907 (DAKO) |
|  |  |  |

**Supplementary Table S2: List of antibodies used in the study.**


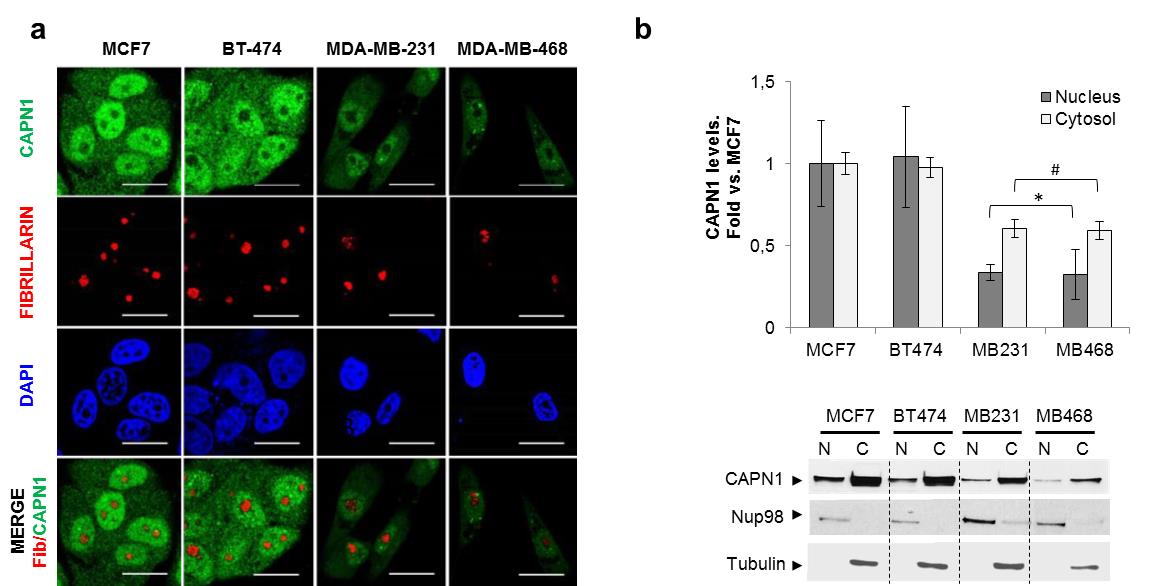


**Supplementary Figure S1. Subcellular distribution of CAPN1 in BCC lines.** a) Immunofluorescence staining of CAPN1 (green), fibrillarin (red), DAPI (blue) and merge in BCC lines. Scale bar 25μm. b) Subcellular distribution of CAPN1 analyzed by western blot in cytosolic (C) and nuclear (N) fractions. Data (n≥3) were quantified, normalized with Nup98 (nuclear fractions) or tubulin (cytosolic fractions) and plotted as mean fold ±SEM vs. MCF-7 cells *p≤0.01. A representative immunoblot is shown. Uncropped images are shown in supplementary figure S14


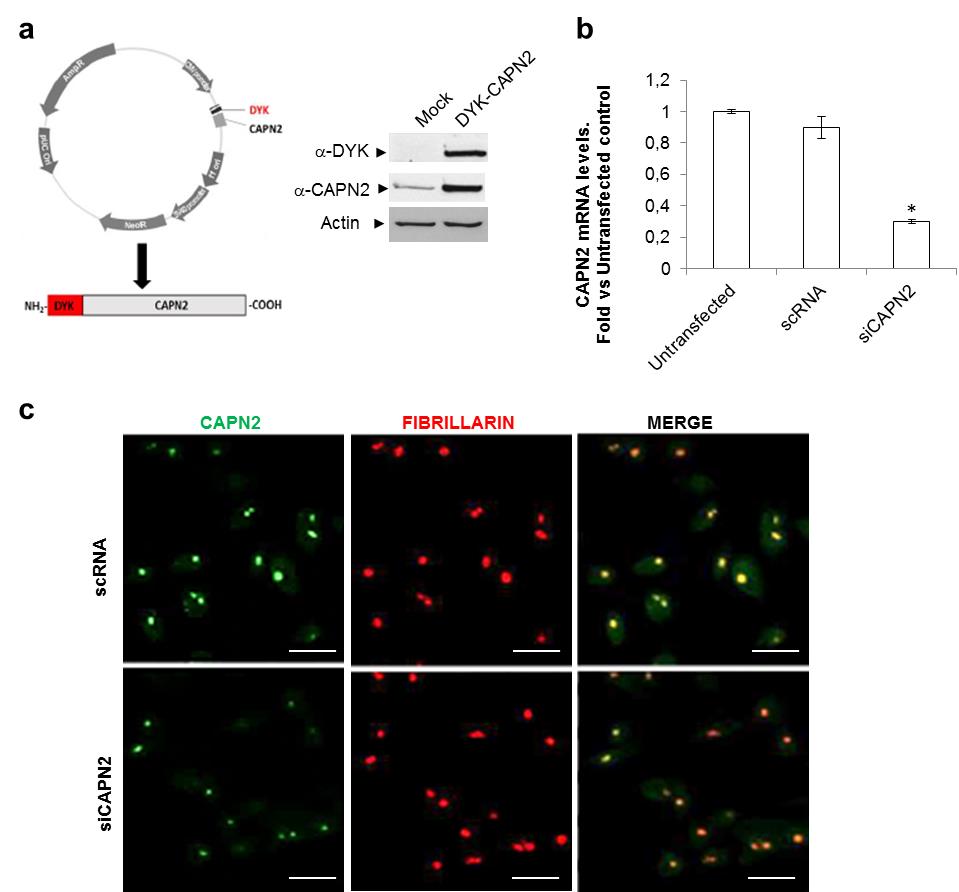


**Supplementary Figure S2. CAPN2 overexpression and downregulation in BCCs.** a) Full-length CAPN2 expression vector. Autoproteolysis of Nt-anchor helix as a mechanism for CAPN2 activation seems to be discarded. The anchor helix becomes disordered and susceptible to proteolysis only after calpain activation [3]. Therefore, DYK epitope was tagged at CAPN2 N-terminal region. MCF-7 cells were transfected with empty vector MSCV (Mock) or DYK- CAPN2 and analyzed by western blot to confirm the efficiency of transfections. Actin was used as loading control. b) RT-qPCR of CAPN2 in MDA-MB-231 cells either untransfected, transfected with scRNA or with siCAPN2. Data (n>6) were normalized by 18S and plotted as mean fold ±SEM vs. untransfected cells *p≤0.01. c) Immunofluorescence analysis of CAPN2 (green) and fibrillarin (red) in scRNA and siCAPN2 transfected MDA-MB-231 cells. Scale bar 25µm. Data n≥6. Yellow and orange indicates high and low colocalization of CAPN2/fibrillarin, respectively


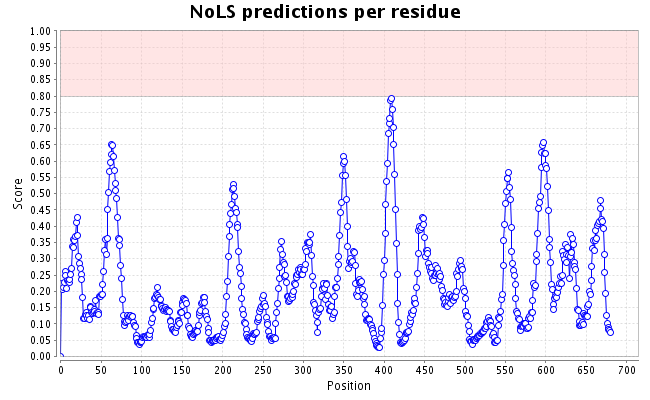


**Supplementary Figure S3. Prediction of NoLS in CAPN2.** Sequence-based search of NoLS motifs in CAPN2 (NP_001739) analyzed by the “Nucleolar localization sequence detector” platform <http://www.compbio.dundee.ac.uk/www-nod/> (Scott, M. S. *et al.* NoD: a nucleolar localization sequence detector for eukaryotic and viral proteins. *BMC Bioinformatics,* **12**, (2011). Threshold score to be recognized as NoLS ≥ 0.8 . A putative NoLS (410-421) was found at the 0.8 score limit


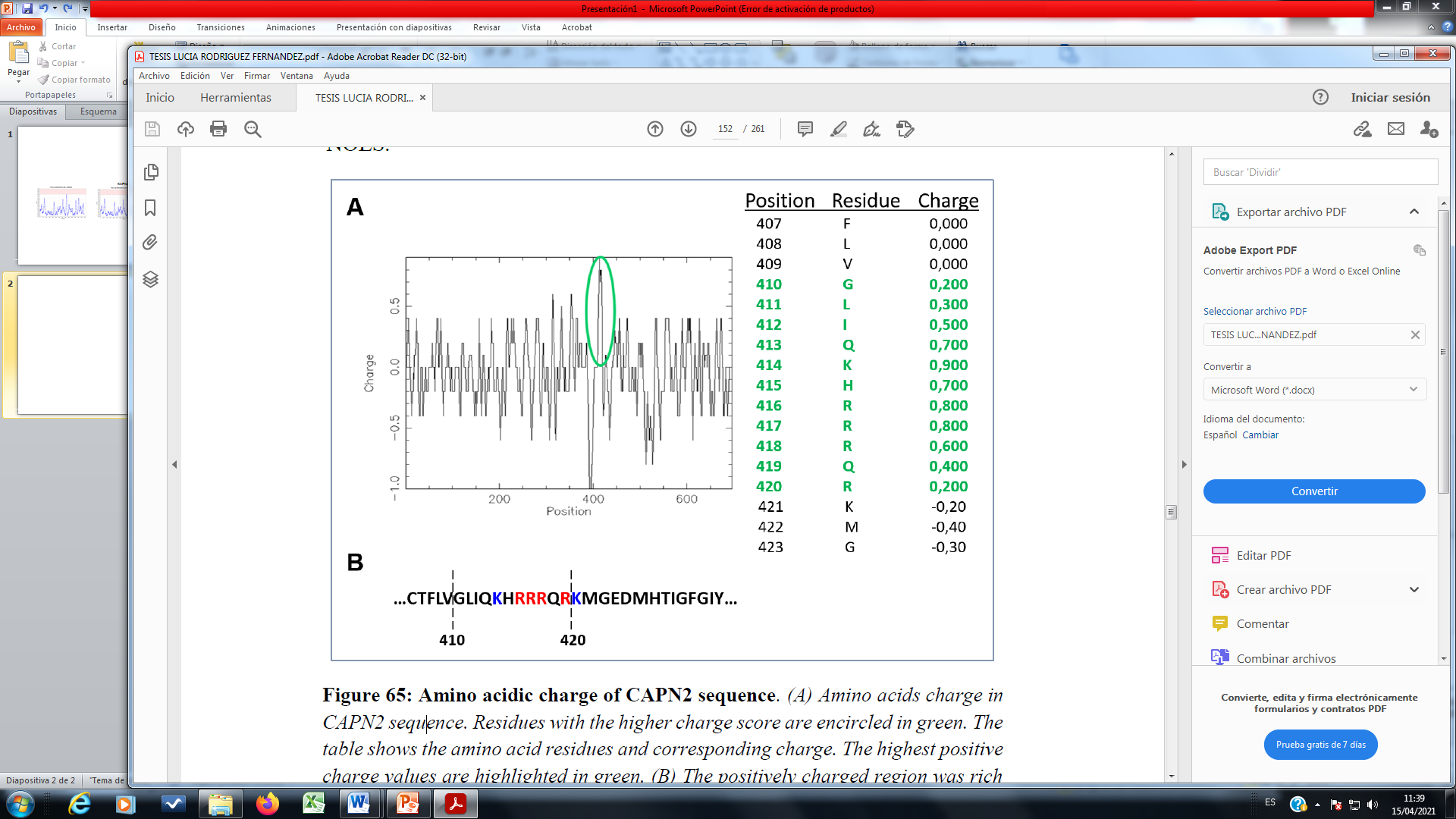


**Supplementary Figure S4. Analysis of positively charged regions in CAPN2 sequence**. a) Positively charged regions in CAPN2 sequence was analyzed by “EMBOSS: charge platform” ([www.bioinformatics.nl/cgi-bin/emboss/charge](http://www.bioinformatics.nl/cgi-bin/emboss/charge)). The algorithm gives the residues 'D' and 'E' a charge of -1, 'K' and 'R' a charge of +1, and the residue 'H' a charge of +0.5. Then it calculates the mean charge across the window, which by default is 5 residues. Residues with the higher charge score are encircled in green. Table shows amino acid positions and the corresponding charges. b) The sequence from positively charged region is shown.


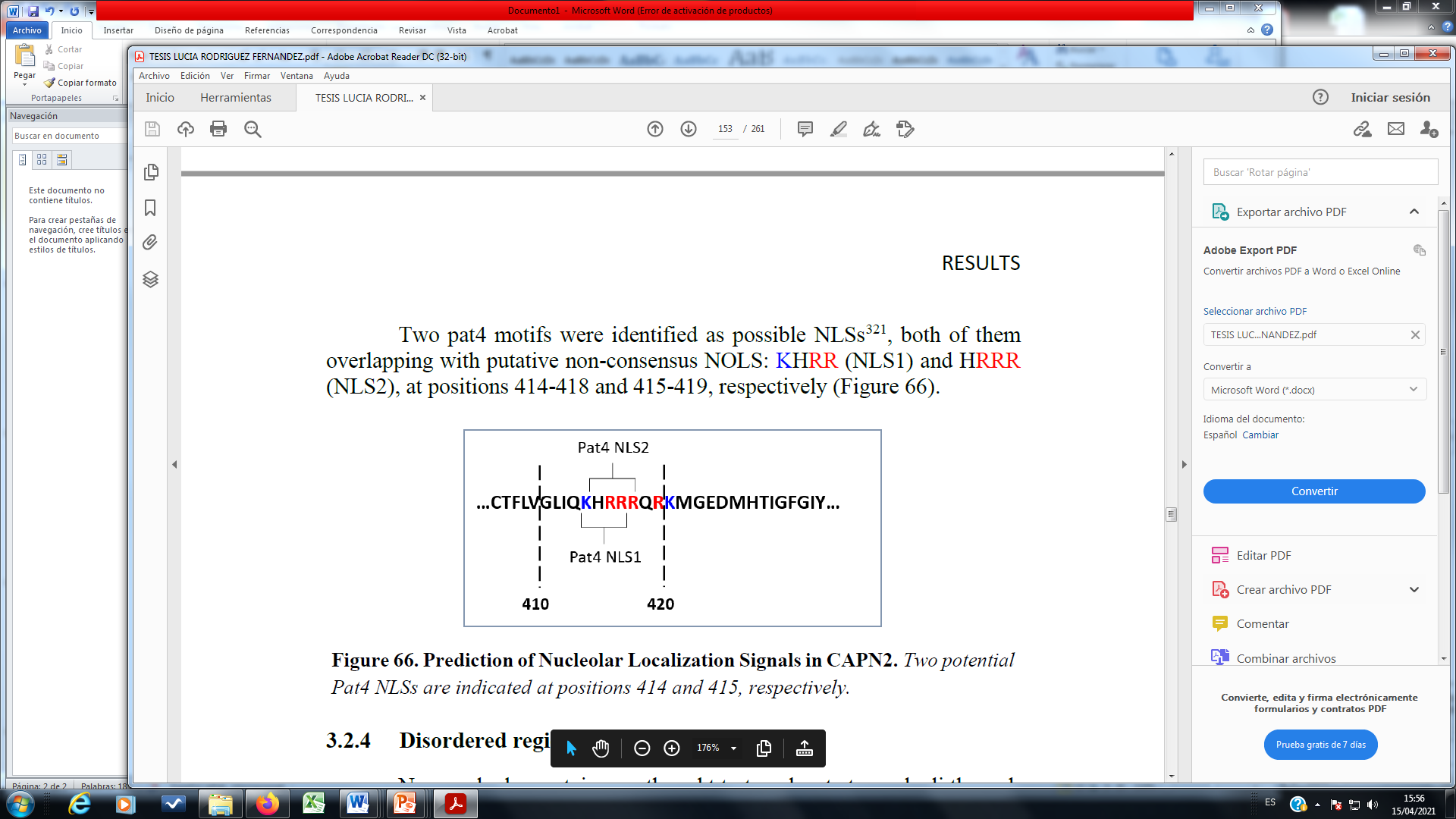


**Supplementary Figure S5. NLS and putative NoLS overlapping regions in CAPN2**. CAPN2, known to localized in nuclei and cytosol, lacks traditional nuclear localization sequences. However, classical definitions of NLSs have been broadened. PSORT II Prediction (<https://psort.hgc.jp/>), a prediction tool designed for detection of protein subcellular localization signals was used to find NLS overlapping sequences (Nakai, K. & Horton, P. PSORT: A program for detecting sorting signals in proteins and predicting their subcellular localization. Trends in Biochemical Sciences, 24, 34–35, 1999).


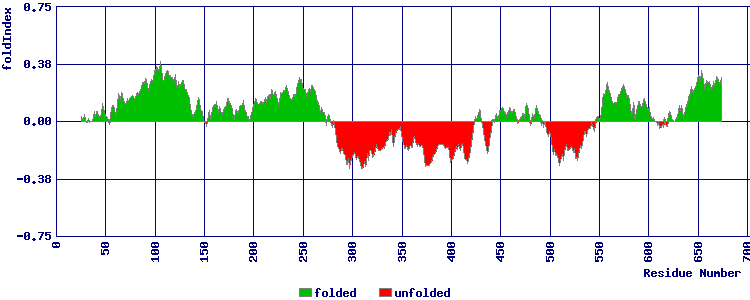


**Supplementary Figure S6 Disordered regions in CAPN2**. Unfolded regions analyzed by Foldindex <https://fold.weizmann.ac.il/fldbin/findex> (Prilusky, J. *et al.* FoldIndex©: A simple tool to predict whether a given protein sequence is intrinsically unfolded. *Bioinformatics,* **21**, 3435–3438 (2005). All positive values represent domains likely to be folded, and negative values represent those likely to be intrinsically unfolded. Four regions were detected as unfolded in CAPN2. The longest disorganized region (282-423; length:142; score: -0.18 ± 0.07) contained the NLSs, the R/K-rich sequence and the putative NoLS.


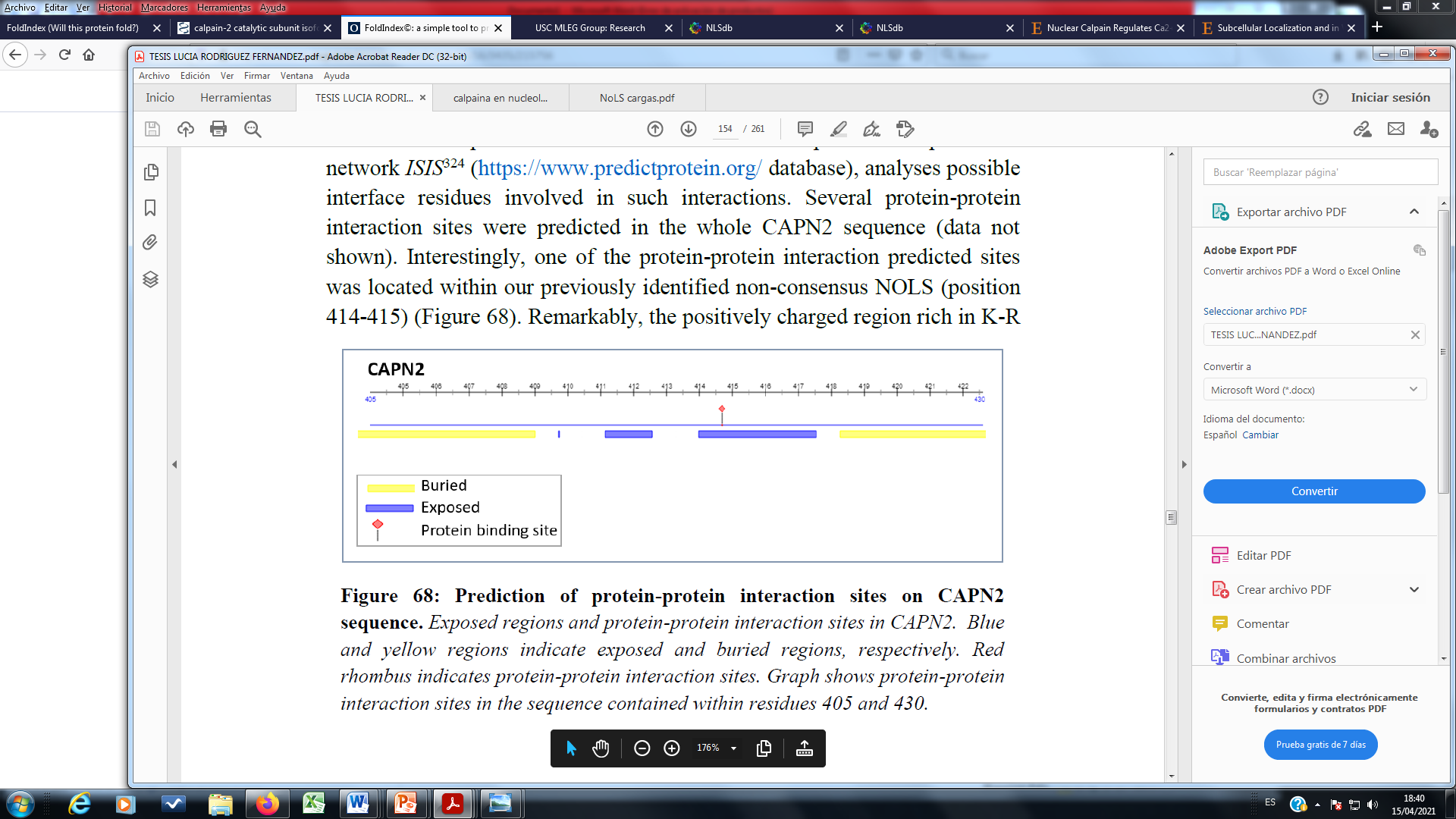


**Supplementary Figure S7. Prediction of protein-protein interaction sites in CAPN2**. Exposed regions and protein-protein binding sites in CAPN2 at [405]-[430] are shown. Blue and yellow indicate exposed and buried regions, respectively. Red rhombus indicates protein-protein interaction sites. Several protein-protein interaction sites were also predicted in the whole CAPN2 sequence (not shown) using the prediction network *ISIS* (https://www.predictprotein.org/database)


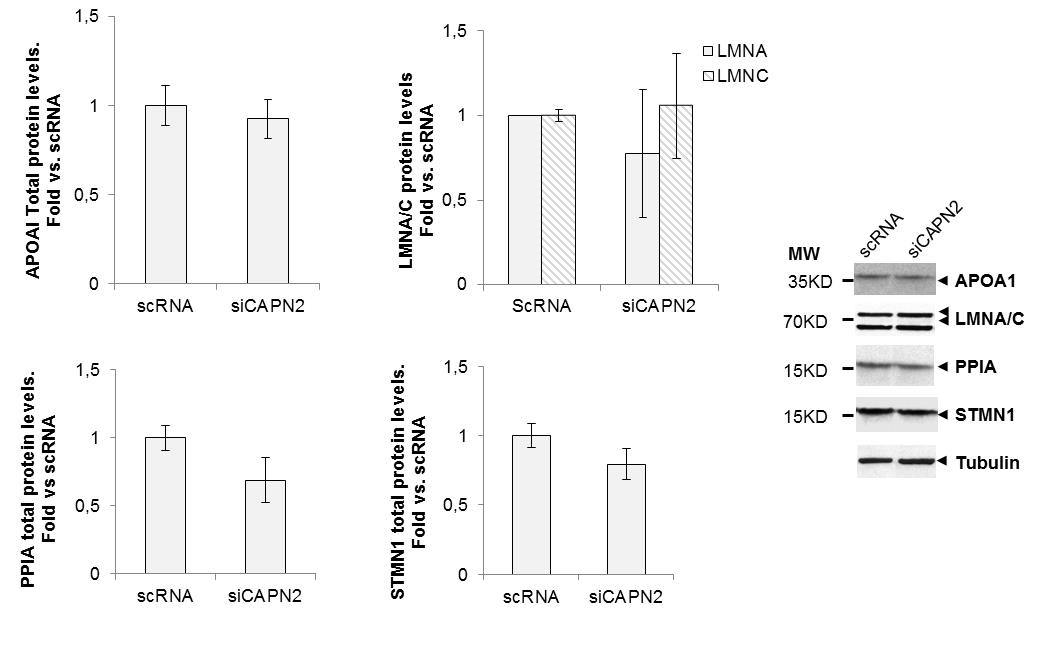


**Supplementary Figure S8. Total protein levels of APOA1, LMNA/C, PPIA and STMN1 upon CAPN2-depletion in MDA-MB-231 cells.** Representative down-regulated (APOA1 and LMNA/C) or up-regulated (PPIA and STMN1) proteins found in nucleoli of CAPN2-depleted cells by 2D-DIGE were selected. Protein levels were analyzed by western blot in whole cell extracts from MDA-MB-231 transfected with scRNA/siCAPN2. Data (n=6) were quantified, normalized (tubulin) and plotted as mean fold ±SEM vs. scRNA. No statistical difference was found.


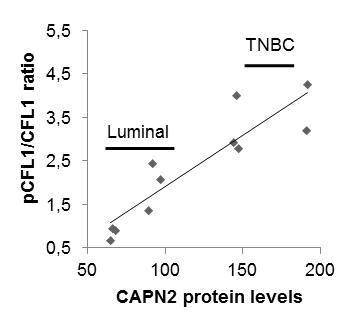


**Supplementary Figure S9. Spearman’s correlation (r) test for CAPN2 protein levels and pCFL1/CFL1 ratio in BCC.** A positive correlation for CAPN2 and pCFL1/CFL1 ratio (r=0.8159, p=0.02) was found in luminal (MCF-7 and BT-474) and TNBC (MDA-MB-231 and MDA-MB-468) cells. The non-parametric Spearman’s correlation rho test was used for correlation analysis between the pair of groups (CAPN2 and pCFL1/CFL1). Both groups of data from luminal and TNBC cells (including triplicates for each cell line) were analyzed. A coefficient correlation (r) value from +0,8 to +1 indicates a strong correlation.

**
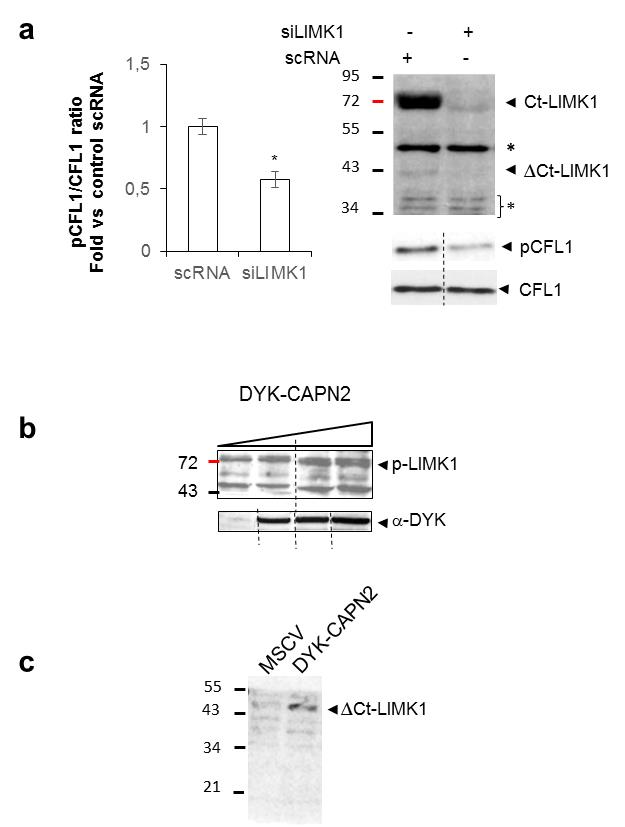
**

**Supplementary Figure S10. LIMK1/pCFL1 and CAPN2/pLIMK1 relationships.** a) PhosphoCFL1 and CFL1 levels analyzed by western blot in total extracts from asynchronous MDA-MB-231 cells transfected with scRNA/siLIMK1. Uncropped images are shown in supplementary figure S14. Data (n≥3) were quantified and pCFL1/CFL1 ratio plotted as mean fold ± SEM. *p≤0.01 vs scRNA. LIMK1 levels were also analyzed to confirm knockdown efficiency. Asterisk indicates unspecific bands detected in both samples. Uncropped image is shown. b) p(Thr-508)LIMK1 levels in MCF7 cells transfected with MSCV or increasing concentrations of DYK-CAPN2 expression-vector analyzed by western blot. Anti-DYK antibody was used to assess transfection efficiency. A ~43K-45KD band was also recognized by anti-p(Thr-508)-LIMK1 antibody. Whether this band represents a fragment of LIMK1 or not is unknown. Uncropped images are shown in supplementary figure S14. C) Western blot of low molecular weight proteins from MSCV and DYK-CAPN2 MCF7-transfected cells analyzed with anti-Ct-LIMK. Membrane was excised and the lower part incubated with anti-Ct-LIMK1. An over-exposed film of full-length image is shown, where a ~43K-45KD band (most likely ∆LIMK1) increases in DYK-CAPN2 overexpressing cells.

**
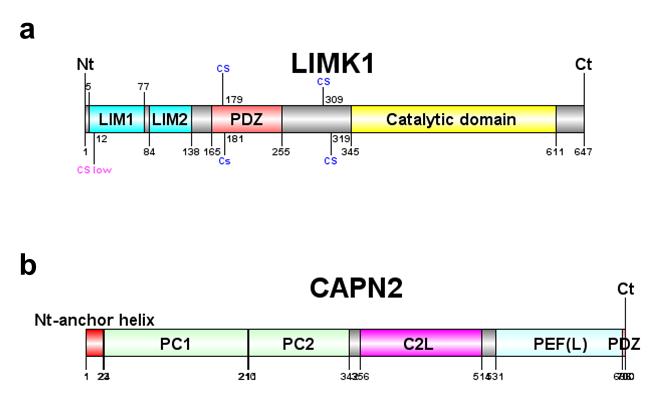
**

**Supplementary Figure S11.** **LIMK1 and CAPN2 structure:** a) LIM domains are connected to a PDZ domain at the N-terminus of LIMK1. PDZ domain is separated from the catalytic domain by a Pro/Ser rich region known to be phosphorylated by several kinases. Predicted CAPN2 cleavage sites in LIMK1 were found using GPS-CCD program [Liu Z, et al. (2011) PLoS ONE 6, e19001]. High threshold was selected for predictions. Only four predicted sites with the highest score (cs-blue) are shown. A low score cleavage site (pink) at N-terminus is also shown. b) CAPN2 structure with PC1 and PC2 catalytic domains followed by C2L and PEF(L). A PDZ domain is present at the C-terminus of CAPN2.


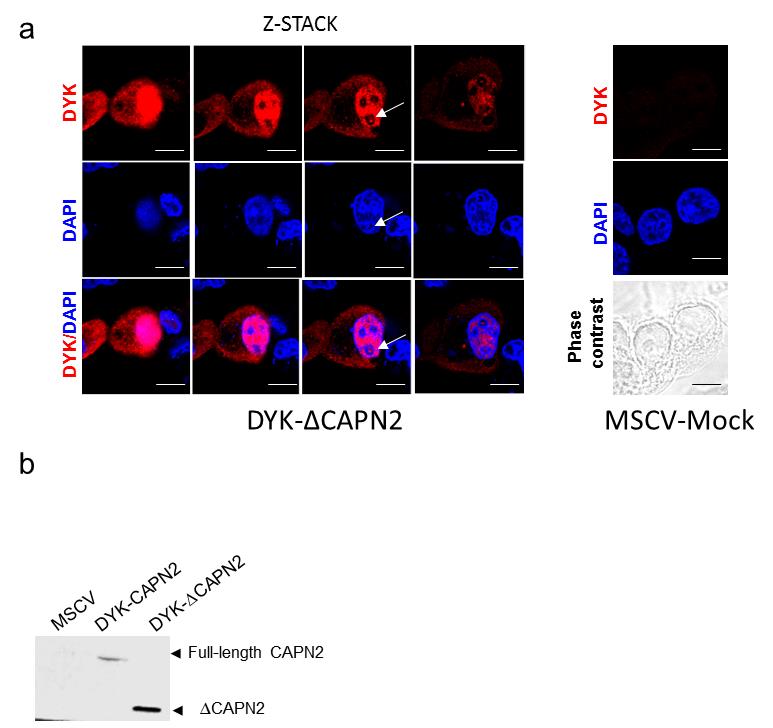


**Supplementary Figure S12. Nucleolar localization of truncated CAPN2.** MCF-7 cells were transfected with empty vector (MSCV), DYK-full-length CAPN2 or DYK-∆CAPN2 expression vectors and analyzed by a) Immunofluorescence staining with anti-DYK antibody (red). Nuclei were counterstained with DAPI. Z-stack images are shown. Arrows indicate the presence of ∆CAPN2 in nucleoli. Scale bars 20 μm. b) Western blot in nucleolar fractions of MCF-7-transfected cells analyzed with anti-DYK antibody. Uncropped image is shown in Supplementary figure S14.

**
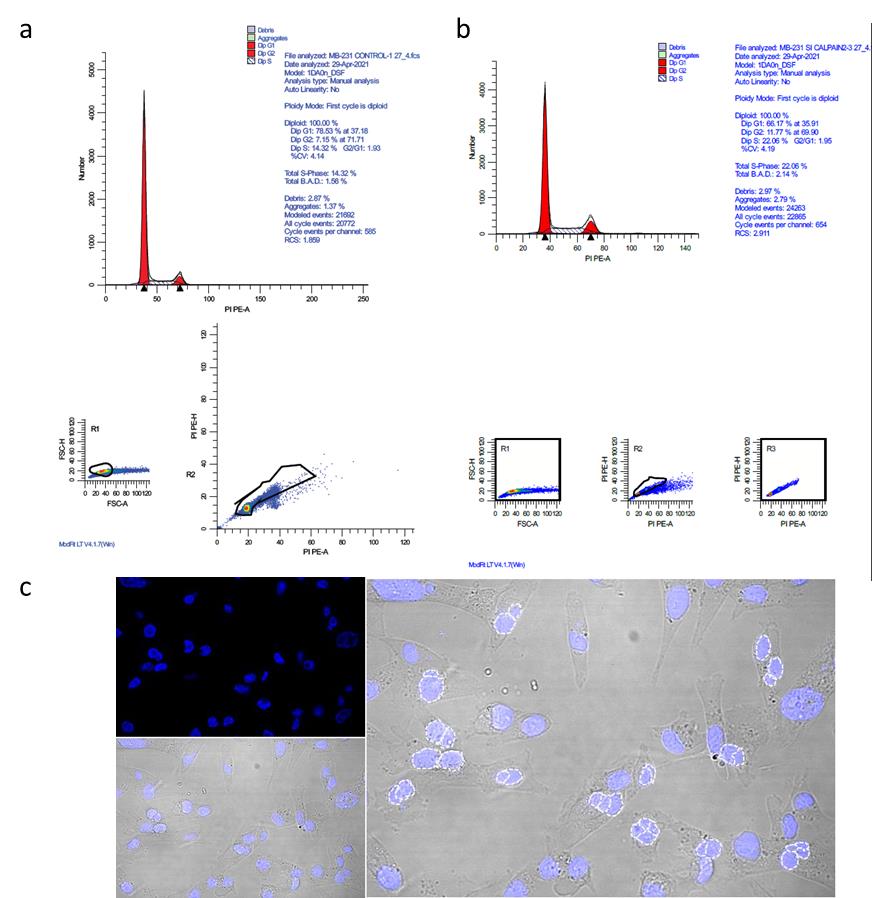

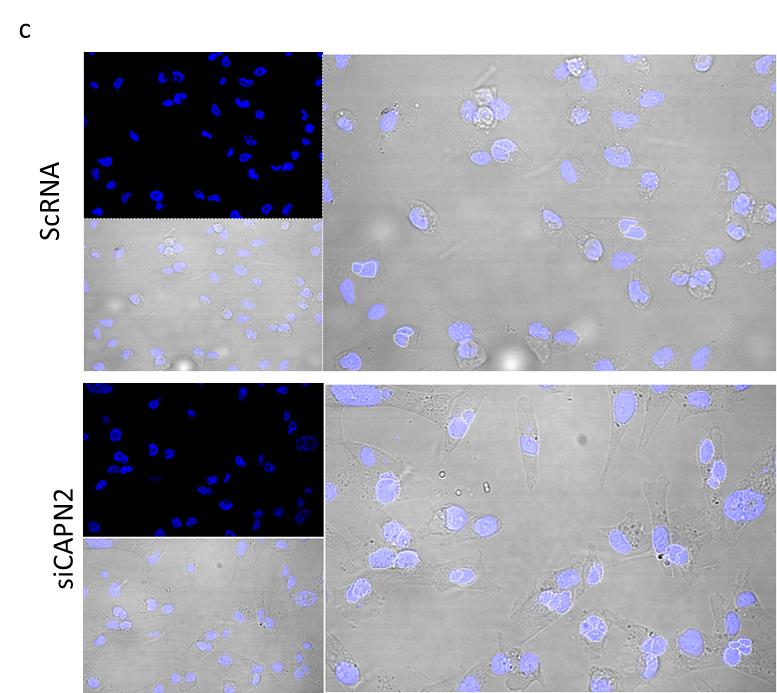
**

**Supplementary Figure S13. Multinucleation of MDA-MB-231 cells upon CAPN2 depletion.** Representative results from cytometric analysis of scRNA (control) a) and siCAPN2 b) transfected MDA-MB-231 cells. Data were obtained from ModFit LT software. c) Nuclei stained with DAPI and merge images of DAPI/phase contrast are shown (left). Magnification of merge images with dashed lines surrounding several nuclei in single cells (right).

**Supplementary Figure S14. Uncropped images shown in figures.** Different lines from the same blot were delineated together in the images shown in the manuscript to follow a homogenous distribution throughout the paper. Lines from different blots were never combined or compared. The cropped images shown in figures are always indicated as a red square on full-length blots.

**
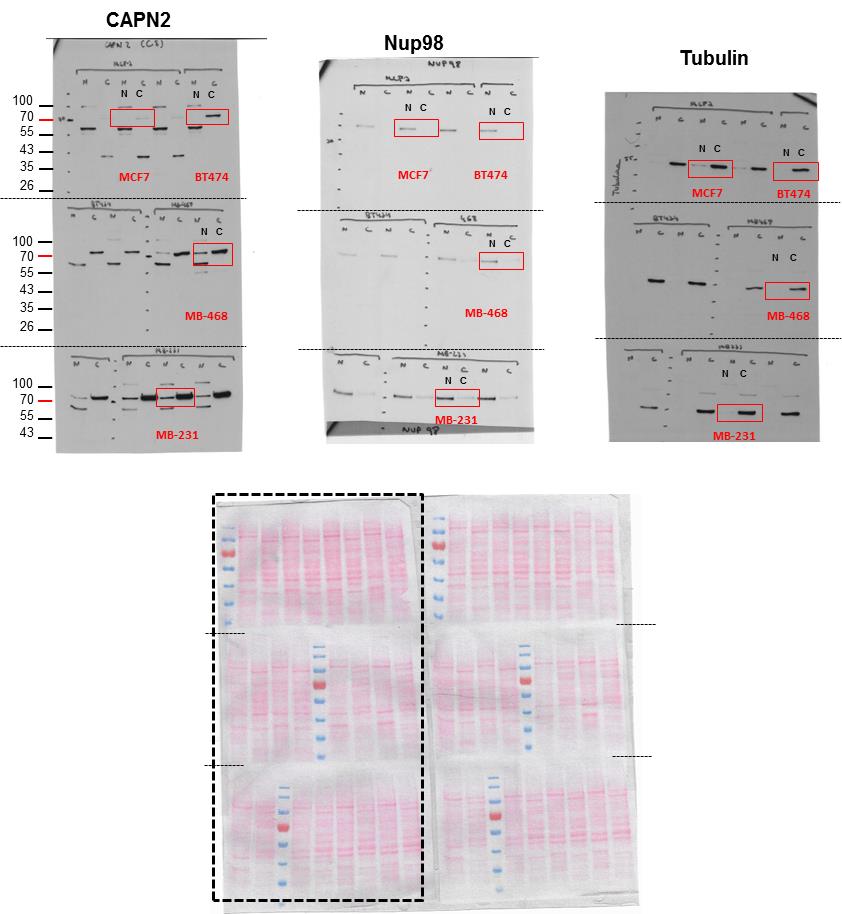
**

**Uncropped Images Figure 1B** Replicates from MCF-7, MDA-BT474, MDA-MB-468 and MDA-MB-231 cell lines were run in three different gels (dotted line), these were electroblotted onto the same membrane (Ponceau stained-membrane is shown) and incubated with the specific primary and HRP-conjugated secondary antibodies. Stripped membranes were re-hybridized with other antibodies.


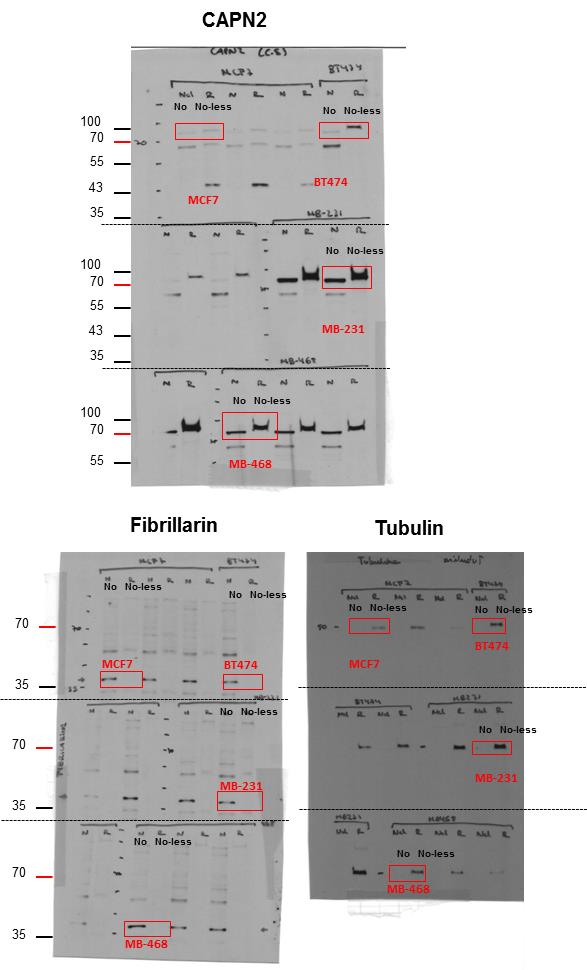


**Uncropped Images Figure 1C.** Nucleolar (No) and Nucleolar-less (No-Less) fractions. Replicates from MCF-7, MDA-BT474, MDA-MB-468 and MDA-MB-231 cell lines were run in three different gels (dotted line), these were electroblotted onto the same membrane and incubated with the specific primary and HRP-conjugated secondary antibodies. Stripped membranes were re-hybridized with other antibodies.

**
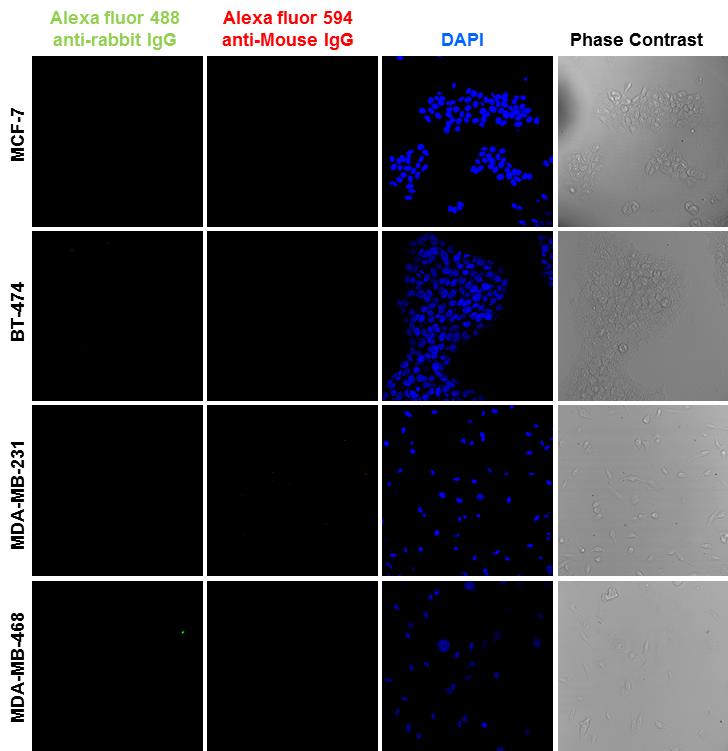
**

**IF negative controls Figure 1A.** Negative controls incubated with all detection reagents except for the primary antibodies. Representative images of the four cell lines are shown.

**
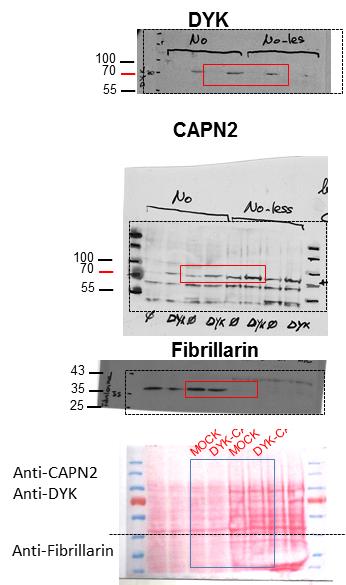
**

**Uncropped images Figure 2B.** Blots were excised and both parts independently hybridized with anti-CAPN2 and anti-Fibrillarin antibodies. The upper part of blot was stripped and re-hybridized with anti-DYK. Full Ponceau-stained membrane is shown. Dotted line in stained-membrane indicates the excision line.


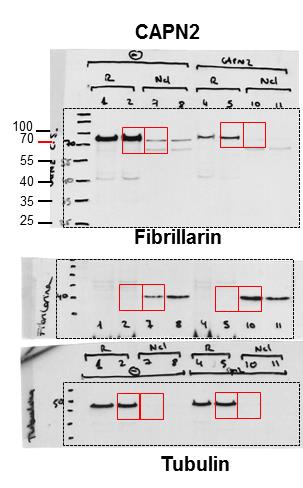


**Uncropped images Figure 2D.** Blots were excised and both parts independently hybridized with anti-Fibrillarin and anti-Tubulin antibodies. Dotted lines indicate the size of membranes.


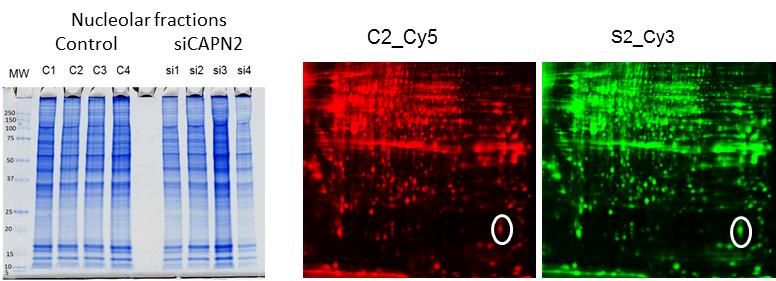


**Uncropped images Figure 3A.** Quality of nucleolar extracts from scRNA (control, C1-C4) and siCAPN2 (S1-S4) transfected cells were analyzed prior to 2D-DIGE. Unmerge 2D-DIGE gels from scRNA (C2) and siCAPN2 (S2) are shown.


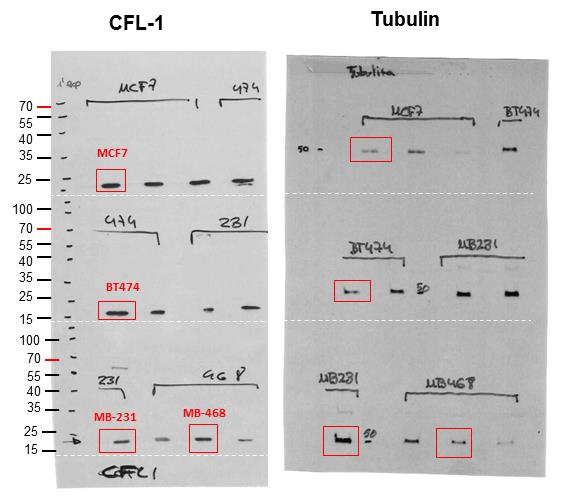


**Uncropped images Figure 3B.** Replicates from MCF-7, MDA-BT474, MDA-MB-468 and MDA-MB-231 cell lines were run in three different gels, these were electroblotted onto the same membrane and incubated with the specific primary and HRP-conjugated secondary antibodies. Stripped membranes were re-hybridized with other antibodies. Dotted white lines indicate the end of each of the three gels.


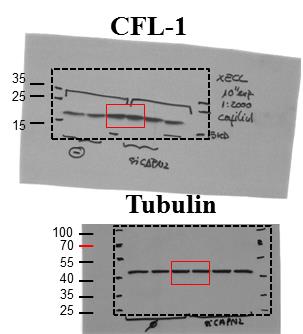


**Uncropped images Figure 3C.** Dotted lines indicate the size of membranes.

**
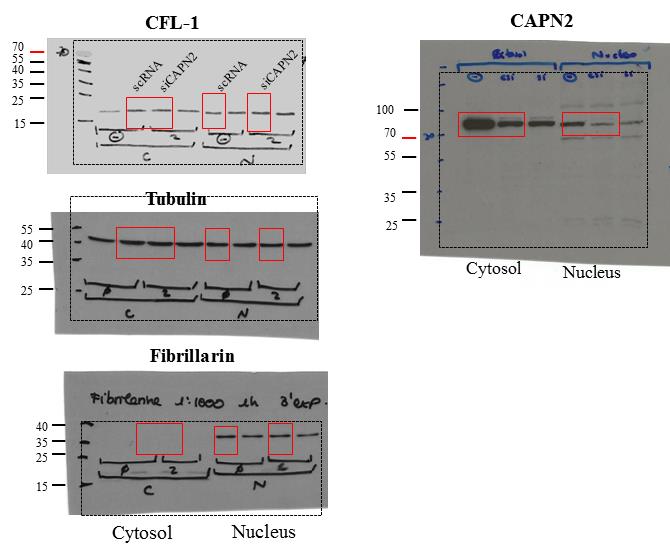
**

**Uncropped images Figure 3E.** Western blot of CFL1 was stripped and reprobed with anti-tubulin and anti-Fibrillarin antibodies. Efficiency of CAPN2 silencing in the same samples was analyzed in a different western blot with anti-CAPN2 antibody. Dotted lines indicate the size of membranes.

**
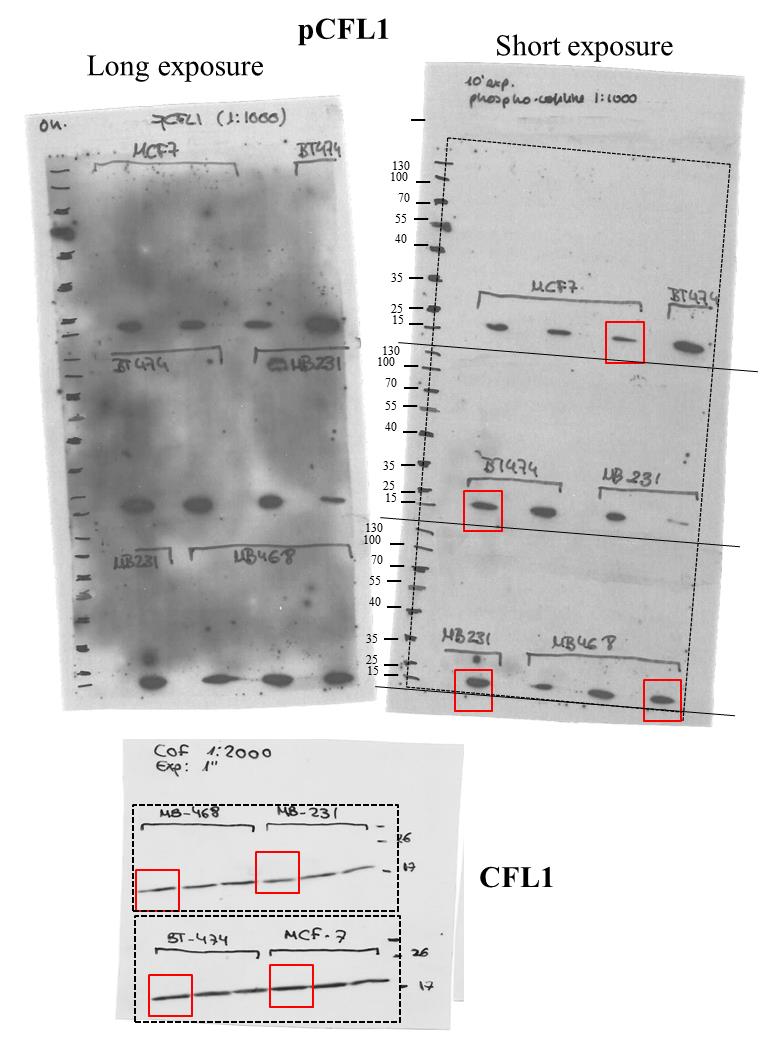
**

**Uncropped images Figure 4A.** Replicates from MCF-7, MDA-BT474, MDA-MB-468 and MDA-MB-231 cell lines were run in three different gels (dotted line), these were electroblotted onto the same membrane and incubated with the specific primary and HRP-conjugated secondary antibodies. Dotted lines indicate the size of gels transferred onto the same membrane.

**
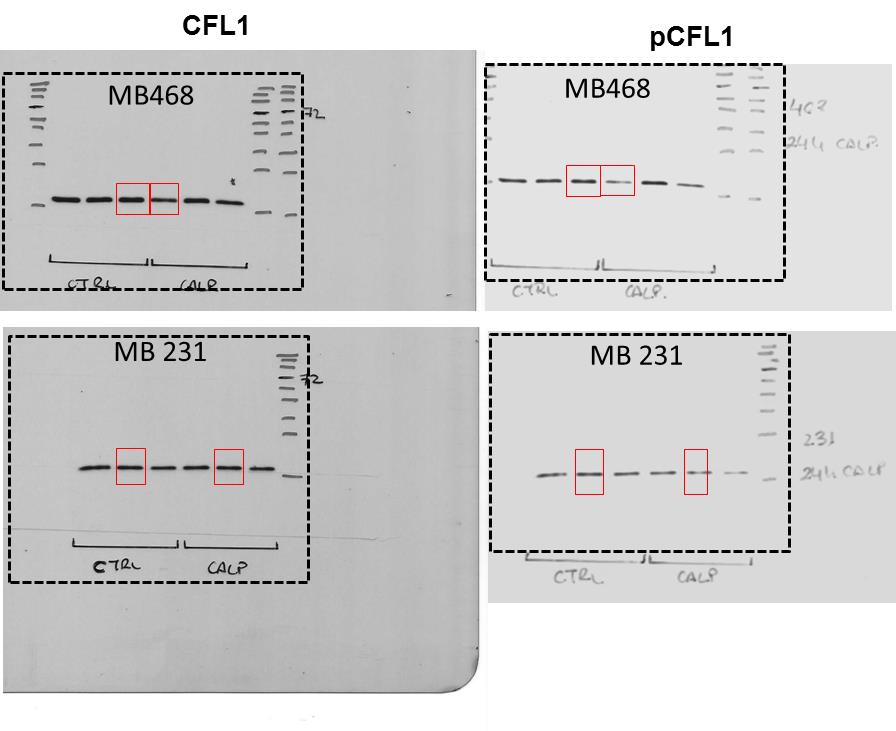
**

**Uncropped images Figure 4B.** Dotted lines indicate the size of membranes.


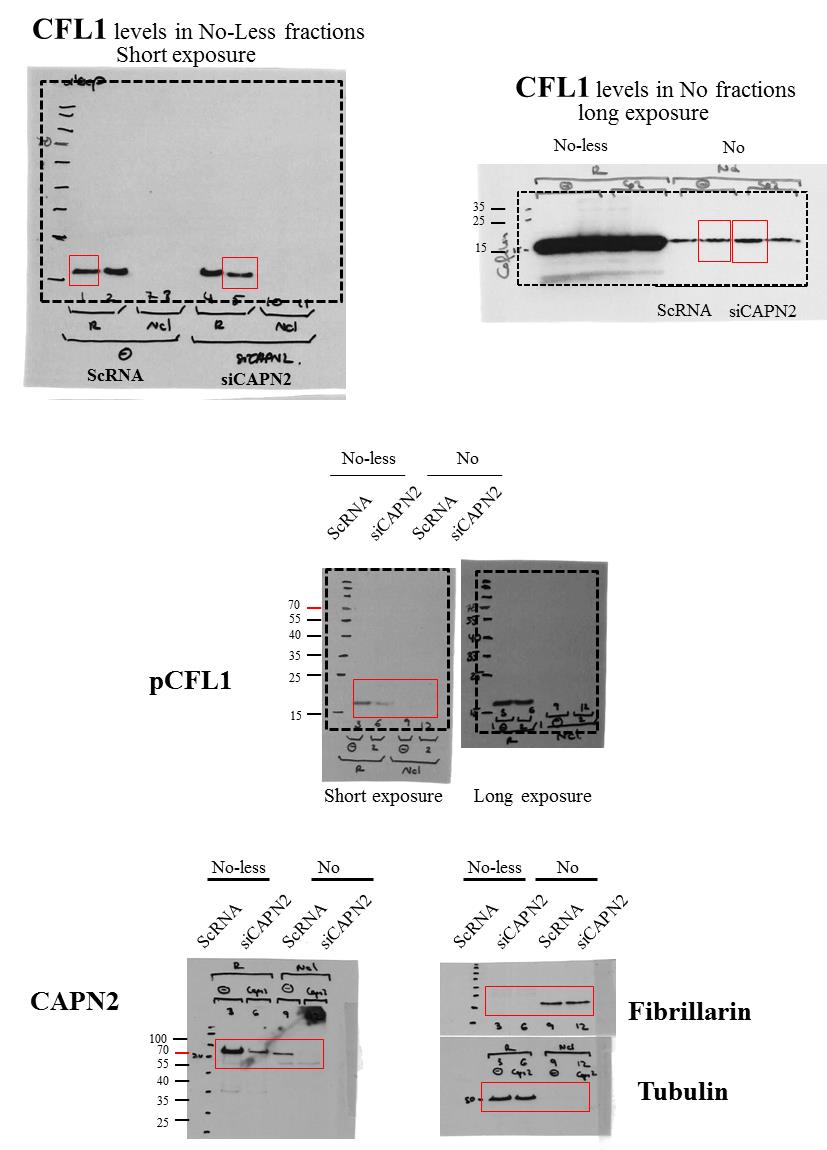


**Uncropped images Figure 4D.**

**
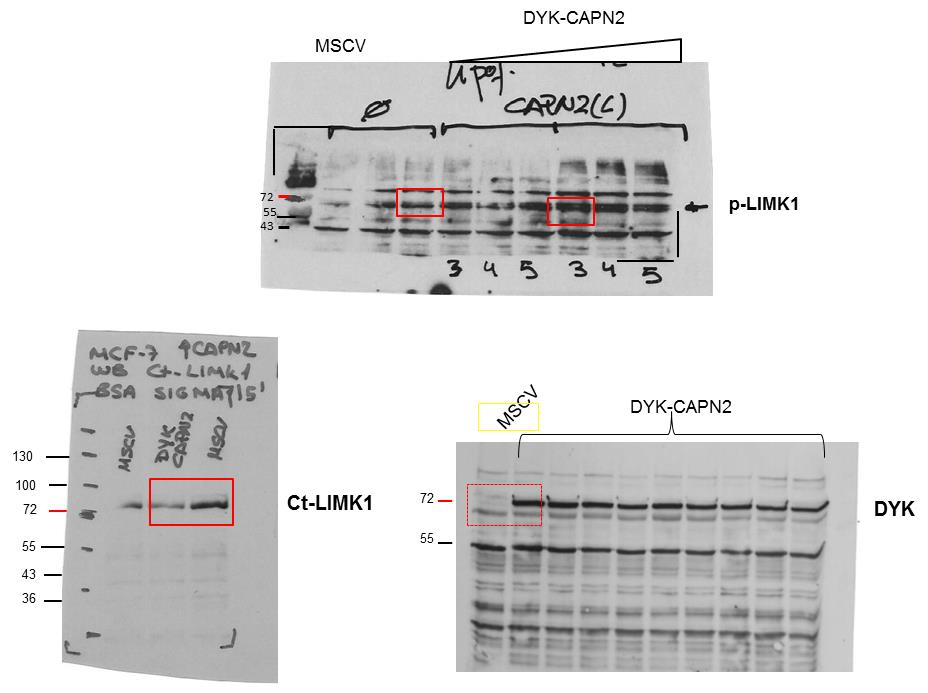
**

**Uncropped images Figure 5A.**

**
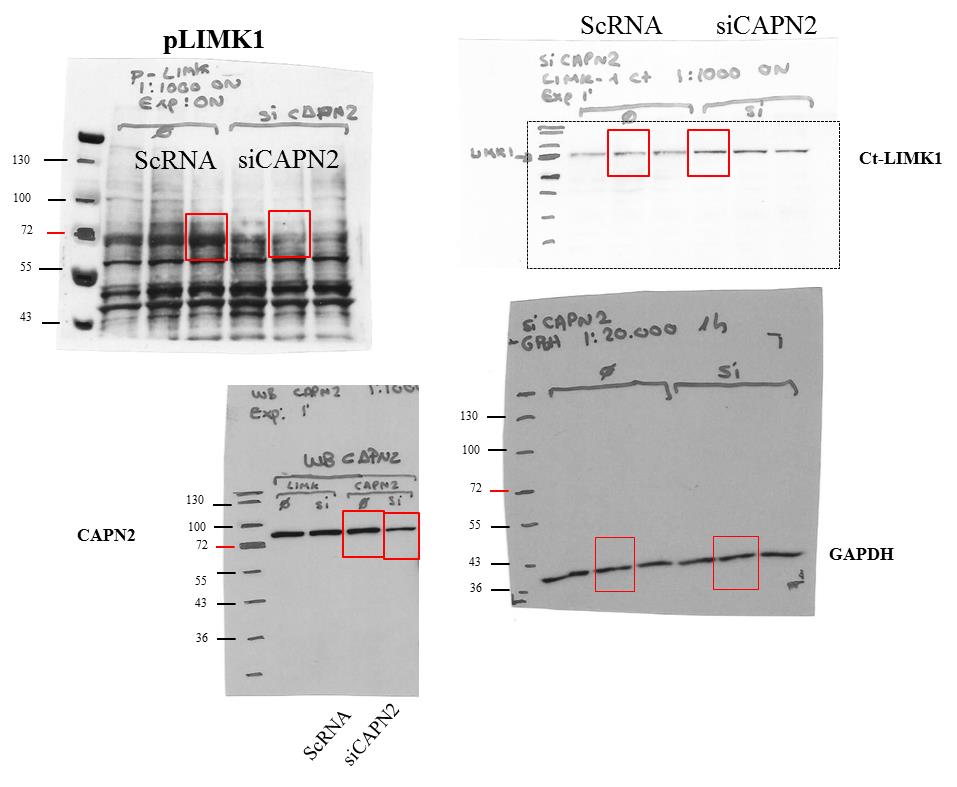
**

**Uncropped images Figure 5B.**

**
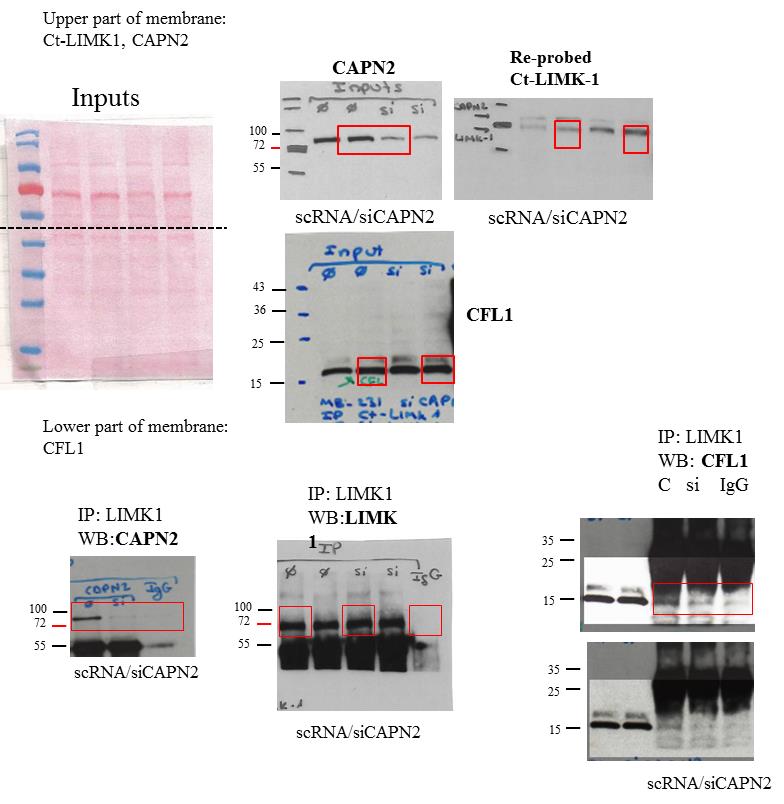
**

**Uncropped images Figure 6A.** Membrane from inputs was excised (dotted line on Ponceau-stained membrane) and both parts independently hybridized with different antibodies.


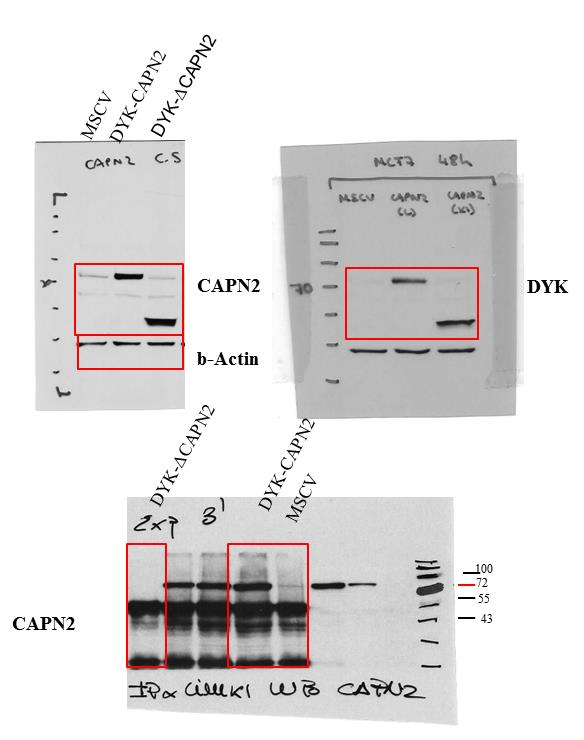


**Uncropped images Figure 6B.**


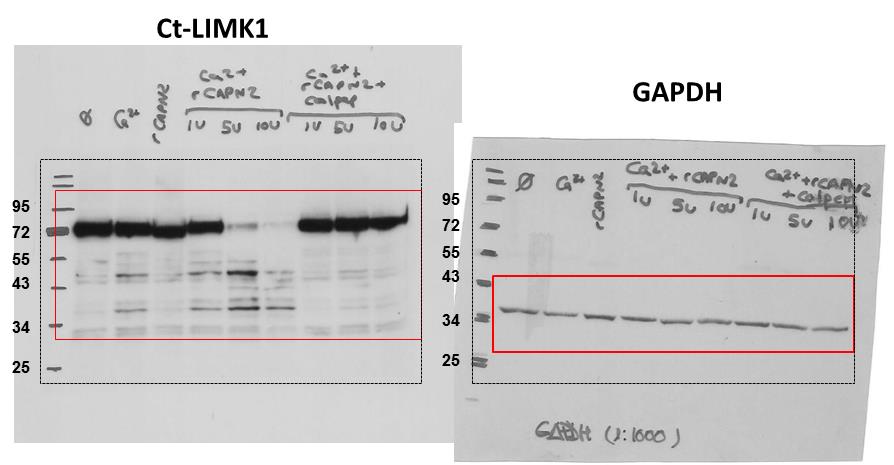


**Uncropped images Figure 7A.**

**
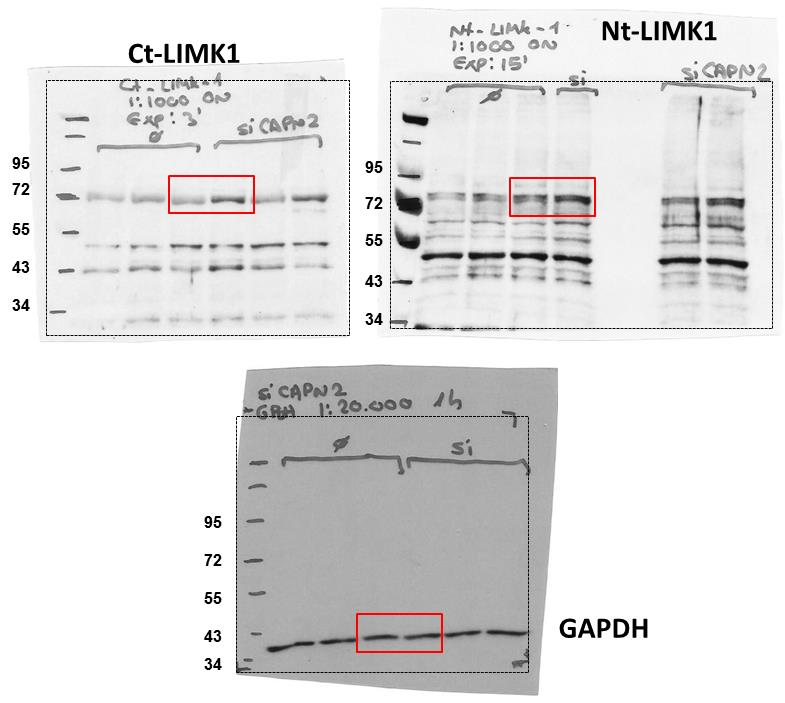
**

**Uncropped images Figure 7B.** Dotted lines indicate the size of membranes.

**
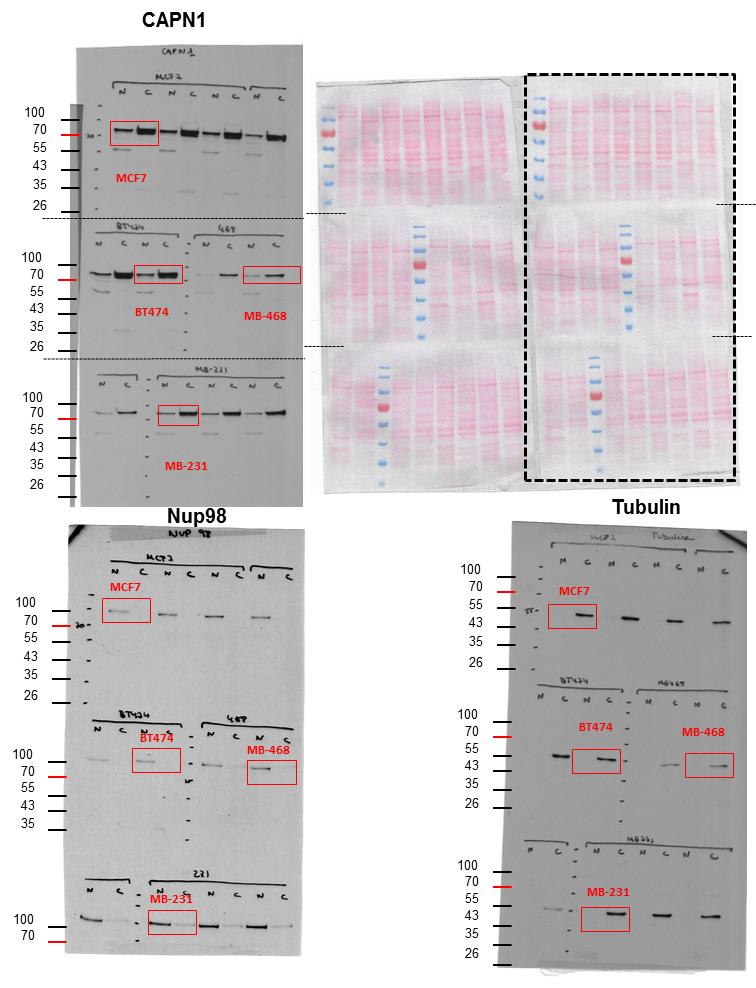
**

**Uncropped images Supplementary Figure S1B** Replicates from MCF-7, MDA-BT474, MDA-MB-468 and MDA-MB-231 cell lines were run in three different gels (dotted line), these were electroblotted onto the same membrane (Ponceau stained-membrane is shown) and incubated with the specific primary and HRP-conjugated secondary antibodies.


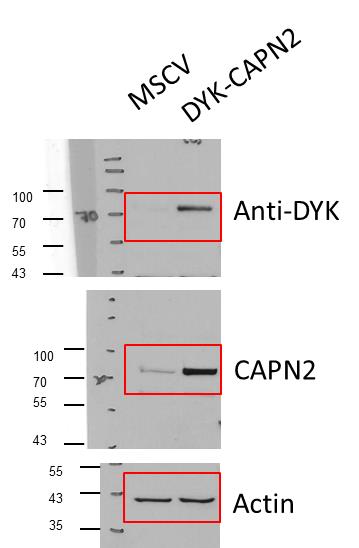


**Uncropped images Supplementary Figure S2A**


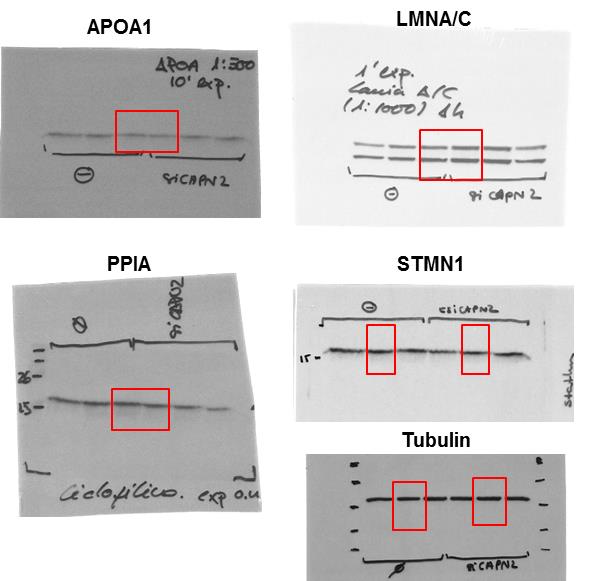


**Uncropped images Supplementary Figure S8**


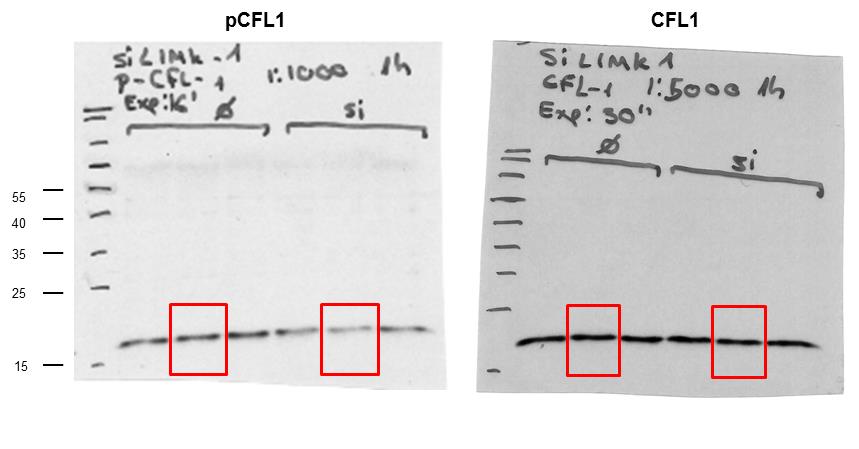


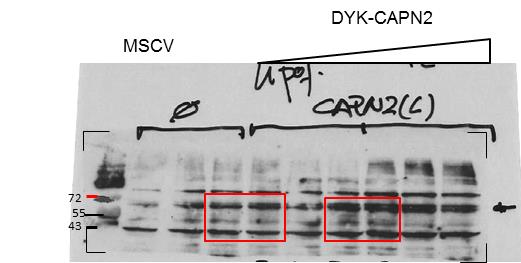


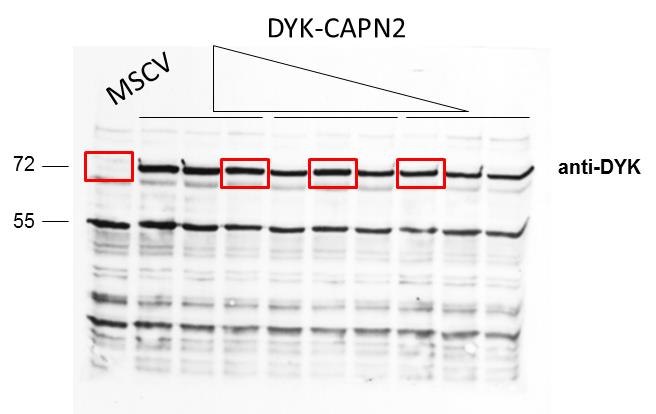


**Uncropped images Supplementary Figure S10A and B.** Membrane incubated with anti-Ct-LIMK1 antibody was excised prior to western blot. Membrane edges are labelled.

**
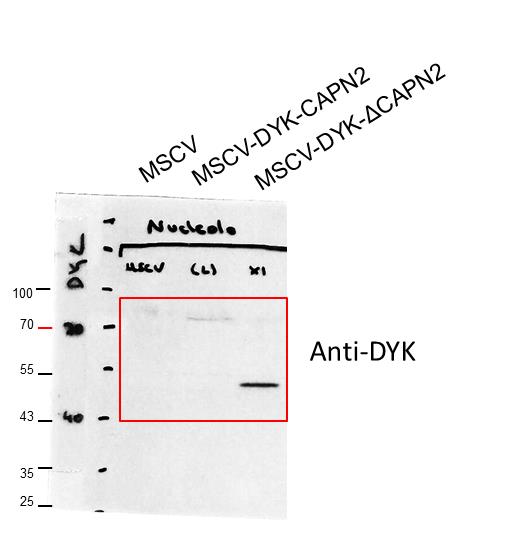
**

**Uncropped images Supplementary Figure S12B**
